# Prevalence, predisposing factors, and turnover intention related to low back pain among health workers in Accra, Ghana

**DOI:** 10.1371/journal.pone.0317582

**Published:** 2025-01-16

**Authors:** Philip Apraku Tawiah, Emmanuel Appiah-Brempong, Paul Okyere, Geoffrey Adu-Fosu, Mary Eyram Ashinyo

**Affiliations:** 1 Department of Occupational and Environmental Health & Safety, School of Public Health, College of Health Sciences, Kwame Nkrumah University of Science and Technology, Kumasi, Ghana; 2 Department of Pharmacognosy and Herbal Medicine, School of Pharmacy, University of Health and Allied Sciences, Ho, Ghana; 3 Department of Health Promotion & Disability Studies, School of Public Health, College of Health Sciences, Kwame Nkrumah University of Science and Technology, Kumasi, Ghana; 4 Physiotherapy Unit, Diagnostic and Rehabilitation Directorate, Ho Teaching Hospital, Ho, Ghana; 5 Department of Quality Assurance–Institutional Care Division, Ghana Health Service Headquarters, Accra, Ghana; 6 Department of Maternal and Child Health, Gilling’s School of Global Public Health, University of North Carolina, Chapel Hill, North Carolina, United States of America; University of Doha for Science & Technology, QATAR

## Abstract

**Background:**

Globally, low back pain (LBP) is responsible for disability among 60.1 million people. Health workers face a higher likelihood of being exposed to LBP compared to employees in the construction and manufacturing sectors. Data on LBP among hospital workers in Ghana are however limited. This study examined the prevalence, predisposing factors and turnover intention related to LBP among health workers in the Greater Accra region of Ghana.

**Methods:**

A multi-centred facility-based cross-sectional study was conducted in the Greater Accra region from January 30 –May 31, 2023. A multi-stage sampling technique was adopted, and the study participants were selected through proportion-to-size simple random sampling. STATA 15 software was used for analysis. Logistic regression analysis was used to determine the factors associated with LBP at a p < 0.05.

**Results:**

A survey was conducted among 607 health workers in 10 public and private hospitals. The prevalence of LBP was 81.6% [95% CI: (78.2%-84.6%)]. Advanced age [AOR = 1.07 (1.00, 1.16)], working for more than 5 days in a week [AOR = 8.14 (2.65, 25.02)], working overtime [AOR = 2.00 (1.16, 3.46)], rarely involved in transferring patients [AOR = 3.22 (1.08, 9.60)], most of the time involved in transferring patients [AOR = 6.95 (2.07, 23.26)], awkward posture during work [(AOR = 2.36 (1.31, 4.25)], perceived understaffing [(AOR = 1.84 (95% CI = 1.04–3.27)], sleep duration ≥ 8 [AOR = 0.54 (0.31, 0.97)] and sitting intermittently at work [AOR = 0.31 (0.12, 0.80)] were factors significantly associated with LBP. A substantial number, 123 (24.9%), occasionally had intention of leaving their jobs.

**Conclusion:**

The study revealed a high proportion of low back pain (LBP), and turnover intention attributed to LBP. Moreover, organizational and occupational factors were found to be significantly associated with LBP. These findings underscore the importance of targeted interventions aimed at reducing the burden of LBP within these specific areas.

## Introduction

Low back pain (LBP) is a highly prevalent and incapacitating condition that affects a significant portion of the population, and it is also a common cause of musculoskeletal disorders [1–3]. LBP remains an overlooked public health problem and responsible for disability among 60.1 million people in the world [2,4] Additionally, it accounts for a number of disability-adjusted life years that is more than those caused by road traffic injuries, communicable and non-communicable diseases [5]. According to the 2010 Global Burden of Disease estimates, LBP ranked among the top ten diseases and injuries with the highest global burden [5]. Furthermore, it is estimated that governments and individuals, especially those in developing countries, spend up to $87.6 billion on medical treatment related to LBP [6].

Worldwide, the prevalence of LBP among adults stands at 40.0%, whilst the incidence rate is about 38.0% on yearly basis [7]. On account of occupational, physical and emotional factors, health workers are at more risk of experiencing LBP than workers of construction and manufacturing industries [8]. Obviously, the nature of work activities carried out by health workers makes them prone to LBP. Moreover, nurses are more exposed compared to other health personnel [9–11]. The main activities include repeated treatment sessions and rehabilitation procedures for patients, manual lifting, handling and transferring of patients, and working in an extreme awkward posture [12]. These tasks are extremely physically demanding, requiring intense workload and effort. Furthermore, in Africa and other low- to middle-income countries, the lack of working aids makes these tasks even more exhausting [13,14]. Some studies have predicted long-term sickness absence as one of the key consequences of LBP [15–17], which may heighten turnover intention among these professionals.

In a recent systematic review and meta-analysis work that included 154 studies, the pooled global lifetime prevalence of LBP among health workers was found to be 54.8% with gender, body mass index, physical exercise, and work-related variables as significant predisposing factors [18]. In another review conducted among nurses working in African clinical settings, prevalence rate of LBP was estimated at 64.1% with the lowest and highest prevalence found to be 44.1% and 82.7%, respectively [19]. Additionally, both lowest and highest prevalence rates were reported from a study conducted in Nigeria, a West African country. With respect to regions, West African region recorded the highest prevalence rate of 68.5% compared to the North and South African regions [19]. These estimates indicate the prevalence of LBP among health workers in West Africa, which includes countries like Ghana. Some studies in Ghana have highlighted the prevalence of turnover intention among health workers, with factors such as inadequate staffing, high workload, and burnout contributing to this phenomenon [20–22]. Additionally, occupational health and safety play a role in employee turnover intention, as job-related stressors like low back pain can impact organizational commitment and job satisfaction, ultimately influencing turnover intentions [23].

Unfortunately, the menace of LBP is understudied in Ghana as evident in the scarcity of studies conducted within the past decade [24]. A recently published scoping review revealed that only two studies had tackled the issue of LBP among health workers in Ghana; and the prevalence rates were 49.5% and 51.2% [25,26]. These two unpublished studies in the review however estimated only the prevalence of LBP without considering the associated predisposing factors. In other words, there is no baseline study that assesses the prevalence and predisposing factors of LBP among health workers in Ghana. This study, therefore, investigated the prevalence, predisposing factors and turnover intention related to LBP among health workers in the Greater Accra region of Ghana.

## Materials and methods

### Study design, participants and setting

This study employed a facility-based analytic cross-sectional research design and a quantitative methodology. The study focused on doctors, nurses, midwives, laboratory staff, physiotherapists, healthcare assistant, orderlies and laundry staff across six public and four private hospitals located in the Greater Accra region of Ghana. These hospitals are major healthcare facilities within their respective districts, providing a range of services including outpatient departments (OPD) services, antenatal and family planning, dental care, eye care, laboratory services, ear-nose-and-throat care, radiology, dermatology services, and surgical procedures. The bed capacity of the hospitals varied from 50 to 500, and the total number of clinical and support staff ranged from 77 to 579. In 2015. The Greater Accra region was home to approximately 30.6% of all healthcare providers, including medical officers, midwives, nurses, and pharmacists, making it the region with the highest density of health workers [27]. In 2021, the Greater Accra Region was also the most populous region in Ghana, with an estimated population of 5,455,692, accounting for approximately 17.7% of the country’s total population [28].

### Sample size determination

The Cochran formulae [29], $N_{o}=\frac{z^{2}pq}{d^{2}}$, was used to determine the sample size for the study. Using z = constant for 95% confidence interval (CI) given as 1.96, p = proportion of the population (39.6%) that were exposed to LBP in a recent study conducted among health workers in Uganda [30], q = (1-p) and d = margin of error estimated as 5%, sample size, *N_o_* was estimated to be 368. After utilizing a design effect of 2.0 as recommended by previous similar studies [31,32], finite correction population formula proposed by Neyman [33,34] and an anticipated 10% non-response rate to the sample size, we arrived at final sample size of 652. However, 607 health workers participated in the study, resulting in a response rate of 93.1%. The main reason for not achieving 100% response rate was lack of monetary compensation.

### Sampling procedure

This study adopted a multi-stage sampling method. The Greater Accra region in Ghana was purposefully chosen, followed by random selection of districts, hospitals, and study participants. The selection of districts, hospitals, and participants was guided by a probability proportional-to-size sampling method. The Greater Accra region comprises 29 districts, including 2 metropolitan areas, 23 municipalities, and 4 districts. For this study, 10 districts, representing over 30.0% of the total, were selected. The sampling frame included 17 major hospitals, of which 10 were randomly chosen for the study. Each district was represented by one major hospital, except in cases districts had two or three major hospitals where one hospital was randomly selected. The selection of major hospitals was influenced by the 2021 annual outpatient department (OPD) attendance data from the District Health Information Management System (DHIMS) [35]. Participants were recruited through stratified random sampling based on their respective profession. The professional groups served as strata, and study participants were randomly selected from them.

### Inclusion and exclusion criteria

The study involved participants including doctors, nurses, midwives, laboratory staff, physiotherapists, healthcare assistants, orderlies and laundry staff who had been working at a hospital for at least one year. Excluded were other healthcare professionals like administrators, radiologists, dieticians, and health students.

### Study questionnaire and data collection

The study questionnaire was purposely designed for the entire study: however, some portions were adapted from National Institute for Occupational Safety and Health, US Centre for Disease Control and Prevention’s Healthcare workers Safety and Health Survey questionnaire [36], and a previous study [30]. The questionnaire comprised of both closed-ended and open-ended questions. The questionnaire was structured into five sections namely: Section I: Respondent’s socio-demographic and lifestyle characteristics; Section II: Occupational factors; Section III: Organizational, behavioural and intervention factors; Section IV: LBP and Section V: Turnover intention with 12, 11, 5, 2 and 2 questions, respectively. To assess the study questionnaire’s validity and reliability, the questionnaire was pre-tested among 60 health workers of the Ho Teaching Hospital. Following the pre-testing was review of questions based on suggestions from study participants, occupational health and safety faculty members, and senior management members of the Ghana Health Service.

The self-administered paper questionnaire in English Language was shared among selected study participants in their various departments at the hospital after a brief interaction with them. Participants were admonished to complete the questionnaire as early as they can; however, participants were given up to the next day to complete the questionnaire. In instances where participants requested for assistance regarding their inability to complete the study questionnaire on their own, research assistants administered the questionnaire in a form of interview. The information on completed paper questionnaire were entered into an earlier developed electronic platform, Open Data Kit [37]. The duration, January 30 –May 31, 2023, was used for recruitment of participants, and data collection.

### Data management and analysis

The data were exported from Open Data Kit electronic platform [37] and imported into STATA SE version 15 (64-bit) statistical analysis software [38] for cleaning and analysis. Participants involved in the pre-testing were excluded from the analysis. Preliminary analysis like frequencies was conducted on all variables to confirm the presence or absence of missing values. Also, skewness and kurtosis tests were performed on quantitative variables to determine their suitability for parametric or non-parametric tests.

Descriptive statistics such as frequencies and percentages were used to summarize categorical variables, whilst median and interquartile range were used for continuous variables. The descriptive statistics of the independent variables (socio-demographic and lifestyle characteristics, occupational factors, organizational, behavioural, and intervention strategies) were presented in the form of tables whereas that of the dependent variable (prevalence of LBP in the past year), and duration of experiencing LBP, and turnover intention after experiencing LBP were presented in pie chart, bar graph and table, respectively. The outcome variable, prevalence of LBP, was evaluated by respondents indicating whether they had experienced LBP within the past year, with ’Yes’ denoting affirmative responses and ’No’ indicating the absence of such experiences. Also, participants were queried regarding the duration of their low back pain experience. Additionally, turnover intention was assessed among study participants who experienced LBP.

A Chi-square, Fisher’s exact and Mann-Whitney U tests were used to determine preliminary associations between prevalence of LBP and independent variables. Additionally, variables significant at a p-value less 0.05 on the above tests were considered and confirmed on the bivariate and multiple logistic regression model. Statistical parameters including crude odds ratio, adjusted odds ratio, 95% CI and p-value were calculated based on a two-sided test.

### Ethical consideration

The study protocol was approved by Committee on Human Research Publication and Ethics (CHRPE) of Kwame Nkrumah University of Science and Technology, Kumasi with an approval reference number, CHRPE/AP/807/22, and the Ghana Health Service Ethics Review Committee with an identity number GHS-ERC:012/03/23. Study participants were briefed on potential risk/benefit, privacy and confidentiality, data storage and usage, voluntary consent/withdrawal, compensation, declaration of conflict of interest and research funding. Finally, participants read and completed a written informed consent form before permitted to take part in the study.

## Results

### Socio-demographic and lifestyle characteristics of health workers

Table 1 presents a summary of health workers sampled from 10 major hospitals in the Greater Accra region of Ghana. Out of the 607 health workers that participated in the study, the majority, 543 (89.3%) and 332 (54.7%) belonged to clinical staff category and nursing profession group, respectively. A little over half of the participants, 312 (51.4%) were within the 30–40-year-old bracket, and the median age was 32 years, with an interquartile range of 28–37 years. The dominant group of the participants, 499 (82.2%) were females, and almost half (49.4%) were married. Most of the study participants, 558 (91.9) had attained tertiary education. A greater portion, 283 (46.6%) had less than 5 years of working experience, and the median working experience was 5 years, with an interquartile range of 3–12 years. Also, 532 (87.6%) worked with public health facilities, and 512 (84.4) were permanent staff. Additionally, a greater number of participants, 493 (81.2) worked for 5 days and below within a week. Besides, more than one-tenth, 100 (16.5) were supervisors. Regarding lifestyle, 308 (50.7%) frequently exercised, and 436 (71.8%) slept for less than 8 hours daily.

**Table 1:** Socio-demographic and lifestyle characteristics of health workers.

Characteristics	*n*	%
***Socio-demographic characteristics***		
**Gender**		
Female	499	82.2
Male	108	17.8
**Age**		
Median (IQR)	32.0	28.0–37.0
Younger than 30	211	34.8
30–39	312	51.4
40–49	68	11.2
50 and older	16	2.6
**Professional category**		
Doctor	41	6.8
Nurse	332	54.7
Midwife	130	21.4
Laboratory staff	34	5.6
Physiotherapist	5	0.8
Orderlies	54	8.9
Laundry staff	2	0.3
Healthcare Assistant	9	1.5
**Type of health worker**		
Clinical staff	543	89.3
Supporting staff	65	10.7
**Marital status**		
Single	295	48.6
Married	300	49.4
Divorced/Separated/widowed	12	2.0
**Highest educational level**		
Primary/secondary	49	8.1
Tertiary	558	91.9
**Type of health facility**		
Private	75	12.4
Public	532	87.6
**Working experience**		
Median (IQR)	5.0	3.0–12.0
Less than 5	283	46.6
5–10	109	18.0
Above 10 years	215	35.4
**Type of employment**		
Contract	95	16.0
Permanent	512	84.0
**Current position**		
No position	473	77.9
Supervisor	100	16.5
Head of Department	34	5.6
**Working days in a typical week**		
5 and below	493	81.2
Above 5	114	18.8
***Lifestyle characteristics***		
**Exercise frequently**		
No	299	49.3
Yes	308	50.7
**Daily hours of sleep**		
Less than 8	436	71.8
8 and above	171	28.2

IQR–Interquartile range.

### Occupational related factors contributing to low back pain

The majority, 310 (51.1%) of study participants worked for overtime. Also, about half (49.9%) were in a mix of day, evening and night shifts, followed by only day shift (46.5%). A substantial number of participants, 232 (38.2%) were assigned on call duties. Also, a dominant number, 570 (93.9%) were full time workers while approximately one-tenth 62 (10.2%) worked in multiple facilities. A greater number of participants, 322 (53.1%) occasionally experienced pressure from work. Additionally, a little over one-third 227 (37.4%) of them sometimes sit for long during work. Most participants, 266 (43.8%) were sometimes engaged in the lifting of heavy objects during their line of work, and a greater number, 213 (35.1%) sometimes found themselves involved in transferring patients. A significant number of participants, 318 (52.4%) worked in awkward posture while 286 (47.1%) sometimes maintain good posture (Table 2).

**Table 2:** Occupational related factors contributing to low back pain.

Characteristics	*N*	%
**Overtime**		
No	297	48.9
Yes	310	51.1
**Type of shift**		
Day only	282	46.5
Evening/swing only	16	2.6
Night only	6	1.0
A mix of day, evening and nights	303	49.9
**On call duties**		
No	375	61.8
Yes	232	38.2
**Type of employment**		
Full time	570	93.9
Part time	37	6.1
**Work in multiple facility**		
No	545	89.8
Yes	62	10.2
**Pressure from work**		
Not at all	28	4.6
Occasionally	322	53.1
Frequently	257	42.3
**Prolong sitting**		
Never	63	10.4
Rarely	132	21.8
Sometimes	227	37.4
Most of the time	141	23.2
Always	44	7.3
**Lifting heavy objects**		
Never	47	7.7
Rarely	148	24.4
Sometimes	266	43.8
Most of the time	108	17.8
Always	38	6.3
**Transferring patients**		
Never	42	6.9
Rarely	89	14.7
Sometimes	213	35.1
Most of the time	184	30.3
Always	79	13.0
**Awkward posture**		
No	289	47.6
Yes	318	52.4
**Maintain good posture**		
Never	28	4.6
Rarely	104	17.1
Sometimes	286	47.1
Most of the time	168	27.7
Always	21	3.5

### Organizational, behavioural and intervention related factors contributing to low back pain

Two-third of participants, 401 (66.1%), mentioned they are understaffed in their department. Also, many of them, 240 (39.5%) used work procedures most of the time. With respect to intervention factors, a good number of participants, 496 (81. %) received training on work machinery, and more than half of them, 311 (51.2%) had also been trained on transport aids. Additionally, most of them, 382 (62.9%) had been given training on good working posture (Table 3).

**Table 3:** Organizational, behavioural and intervention related factors contributing to low back pain.

Characteristics	*N*	%
***Organizational factors***		
**Understaffed**		
No	206	33.9
Yes	401	66.1
***Behavioural factors***		
**Use of work procedures**		
Never	13	2.1
Rarely	44	7.3
Sometimes	95	15.7
Most of the time	240	39.5
Always	215	35.4
***Interventional factors***		
**Trained on work machinery**		
No	111	18.3
Yes	496	81.7
**Trained on transport aids**		
No	296	48.8
Yes	311	51.2
**Trained on good working posture**		
No	225	37.1
Yes	382	62.9

### Proportion of LBP among Health Workers

**Fig 1:**
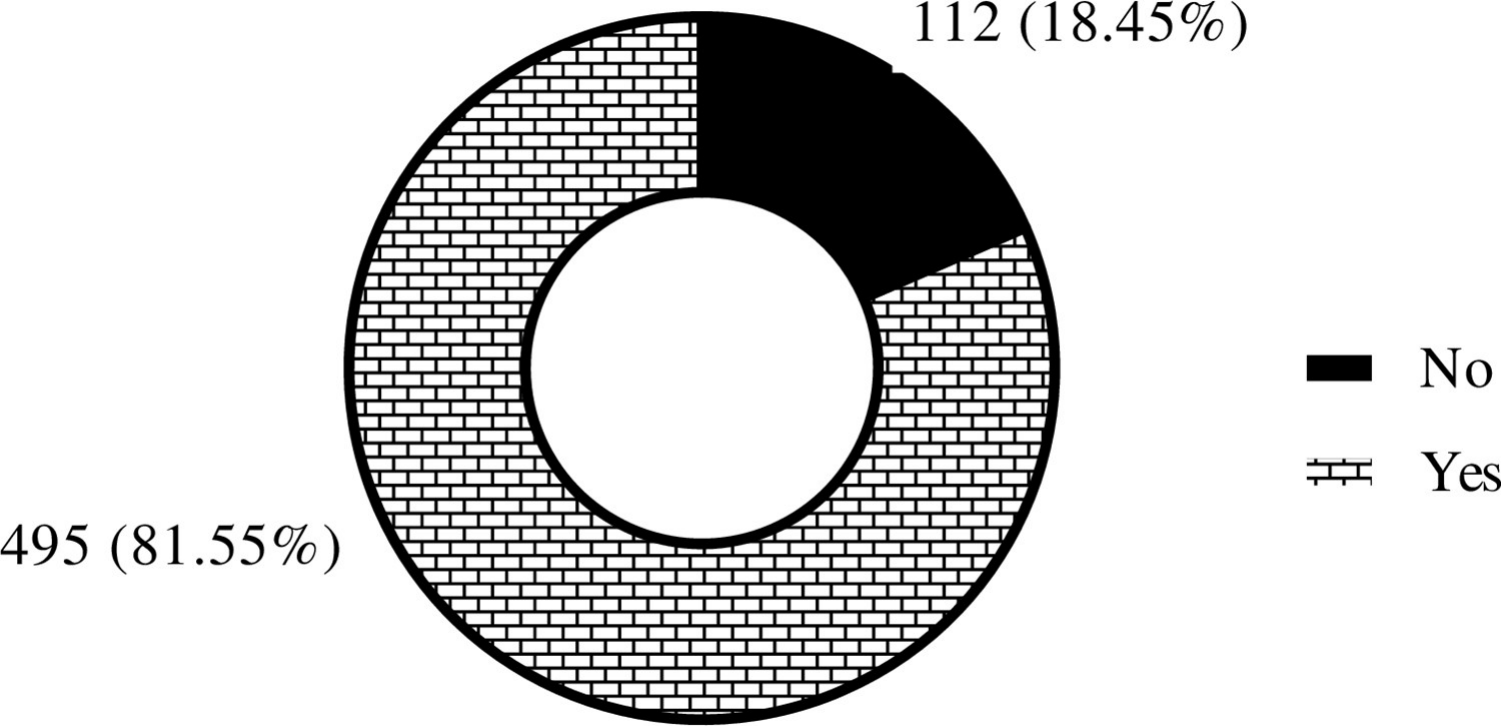
Proportion of LBP among health workers.

**Fig 2:**
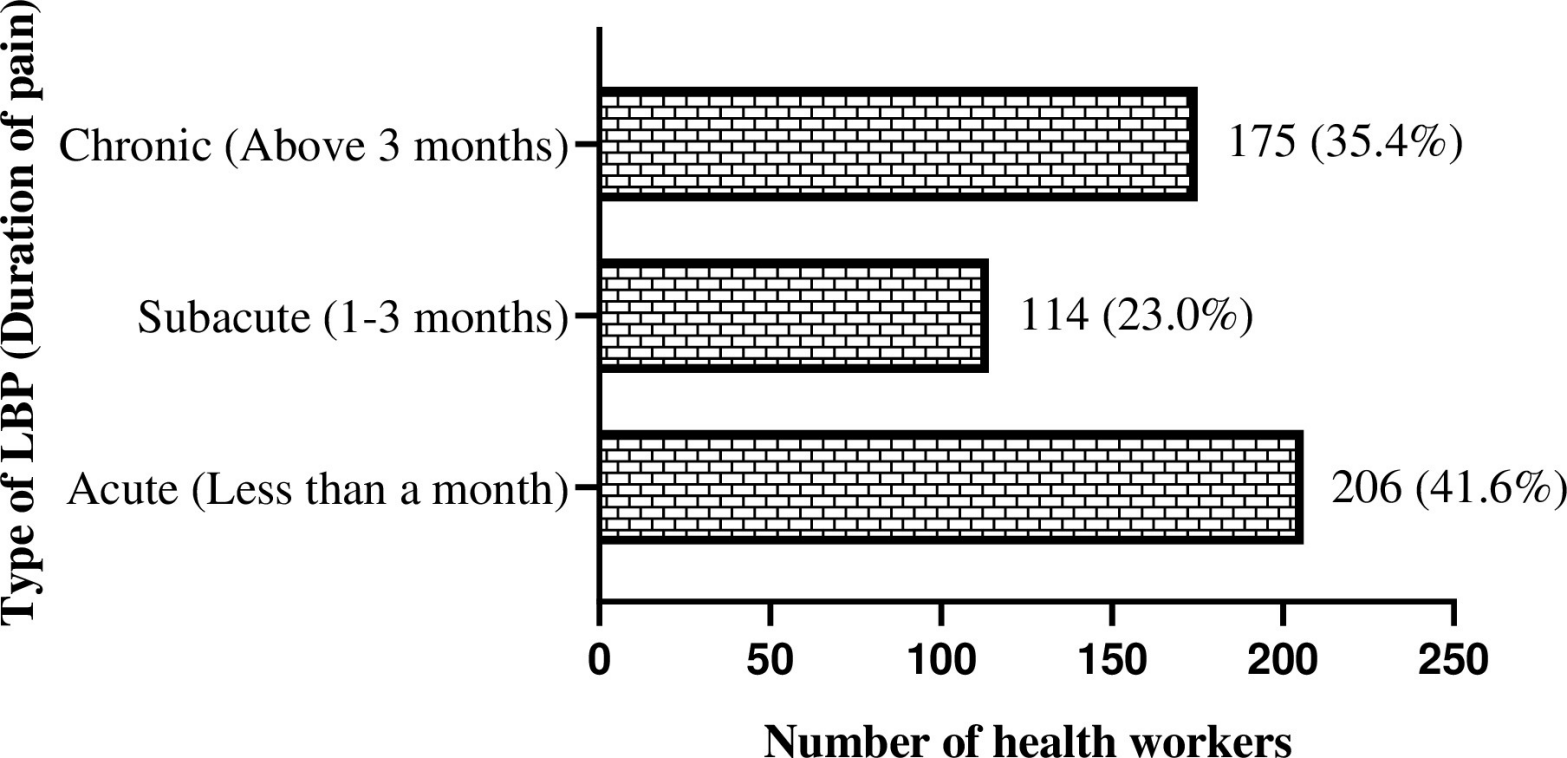
Type of LBP experienced by health workers.

As depicted in Fig 1, a huge number of participants, 495 (81.6%) [95% CI: (78.2%-84.6%)] experienced LBP in the past year. The majority of these cases of LBP, 206 (41.6%) lasted for less than a month (acute LBP) while a substantial number, 175 (35.4%) persisted for more than 3 months (chronic LBP) (Fig 2).

### Socio-demographic and lifestyle characteristics influencing LBP

A significant association was found between age (t = - 3.57, p < 0.001), working experience (t = - 2.41, p = 0.015), type of employment (***χ*2** = 12.90, p < 0.001), number of working days in a typical week (***χ*2** = 20.83, p < 0.001), and prevalence of LBP (Table 4). Regarding lifestyle characteristics, frequency of exercise (***χ*2** = 4.53, p = 0.033) and daily hours of sleep (***χ*2** = 12.91, p < 0.001) were found to be significantly related to prevalence of LBP (Table 4).

**Table 4:** Socio-demographic and lifestyle characteristics influencing LBP.

Characteristics	*N*	LBP		*χ*2	p
		No	Yes		
**Gender**				0.71	0.401
Female	499	89 (17.84)	410 (82.16)		
Male	108	23 (21.30)	85 (78.70)		
**Age**				- 3.57	**<** 0.001*^b^
Median (IQR)	32.0 (28.0–37.0)	30.0 (26.0–35.0)	32.0 (28.0–37.0)		
**Type of health worker**				1.03	0.311
Clinical staff	543	103 (19.00)	439 (81.00)		
Supporting staff	65	9 (13.85)	56 (86.55)		
**Marital status**				2.87	0.264^a^
Single	295	57 (19.32)	238 (80.68)		
Married	300	55 (18.33)	245 (81.67)		
Divorced/separated/widowed	12	0 (0.00)	12 (100.00)		
**Highest educational level**				2.41	0.176
Primary/secondary	49	5 (10.20)	44 (89.90)		
Tertiary	558	107 (19.45)	451 (80.82)		
**Type of health facility**				1.01	0.315
Private	75	17 (22.67)	58 (77.33)		
Public	532	95 (17.86)	437 (82.14)		
**Working experience**				- 2.41	0.015*^b^
Median (IQR)	5.0 (3.0–12.0)	4.0 (2.0–10.0)	5.0 (3.0–12.0)		
**Type of employment**				12.90	< 0.001*
Contract	95	30 (31.58)	65 (68.42)		
Permanent	512	82 (16.02)	430 (83.98)		
**Current position**				2.30	0.317^a^
No position	473	89 (18.82)	384 (81.18)		
Supervisor	100	20 (20.00)	80 (80.00)		
Head of Department	34	3 (8.82)	31 (91.18)		
**Exercise frequently**				4.53	0.033*
No	299	45 (15.05)	254 (84.95)		
Yes	308	67 (21.75)	241 (78.25)		
**Daily hours of sleep**				12.91	< 0.001*
Less than 8	436	65 (14.91)	371 (85.09)		
8 and above	171	47 (27.49)	124 (72.51)		
**Working days in a week**				20.83	< 0.001*^a^
5 and below	493	108 (21.91)	385 (78.09)		
Above 5	114	4 (3.51)	110 (96.49)		

* Significant variable (p < 0.05)

^a^ p-values calculated from Fishers’ exact test.

^b^ p-values calculated from Mann-Whitney U test; IQR–Interquartile range.

### Occupational factors influencing LBP

As shown in Table 5, a significant association was revealed between overtime (***χ*2** = 16.15, p < 0.001), on call duties (***χ*2** = 4.46, p = 0.035), and prevalence of LBP. Additionally, prolong sitting (***χ*2** = 18.90, p = 0.001), awkward posture (***χ*2** = 38.66, p < 0.001), and maintaining good posture (***χ*2** = 21.95, p < 0.001) were found to be significantly related to prevalence of LBP. Also, a significant relationship was shown between lifting heavy objects (***χ*2** = 14.54, p = 0.006), transferring patients (***χ*2** = 29.23, p < 0.001), and prevalence of LBP. Again, pressure from work (***χ*2** = 29.23, p < 0.001) was found to be associated with prevalence of LBP.

**Table 5:** Occupational factors influencing LBP.

Characteristics	*N*	LBP		*χ*2	p
		No	Yes		
**Overtime**				16.15	< 0.001*
No	297	74 (24.92)	223 (75.08)		
Yes	310	38 (12.26)	272 (87.74)		
**Type of shift**				5.17	0.169a
Day only	282	54 (19.15)	228 (80.85)		
Evening/swing only	16	0 (0.00)	16 (100.00)		
Night only	6	0 (0.00)	6 (100.00)		
A mix of day, evening and nights	303	58 (19.14)	245 (80.86)		
**On call duties**				4.46	0.035*
No	375	79 (21.07)	296 (78.93)		
Yes	232	33 (14.22)	199 (85.55)		
**Type of employment**				0.90	0.342
Full time	570	103 (18.07)	467 (81.93)		
Part time	37	9 (24.32)	28 (75.68)		
**Work in multiple facility**				0.29	0.590
No	545	99 (18.17)	446 (81.83)		
Yes	62	13 (20.97)	49 (79.03)		
**Pressure from work**				16.93	< 0.001*
Not at all	28	7 (25.00)	21 (75.00)		
Occasionally	322	77 (23.91)	245 (76.09		
Frequently	257	28 (10.89)	229 (89.11)		
**Prolong sitting**				18.90	0.001*^a^
Never	63	11 (17.46)	52 (82.54)		
Rarely	132	13 (9.85)	119 (90.15)		
Sometimes	227	60 (26.43)	167 (73.57)		
Most of the time	141	24 (17.02)	117 (82.98)		
Always	44	4 (9.09)	40 (90.91)		
**Lifting heavy objects**				14.54	0.006*^a^
Never	47	16 (34.04)	31 (65.96)		
Rarely	148	27 (18.24)	121 (81.76)		
Sometimes	266	53 (19.92)	213 (80.04)		
Most of the time	108	14 (12.96)	94 (87.04)		
Always	38	2 (5.26)	36 (94.74)		
**Transferring patients**				29.23	< 0.001*
Never	42	16 (38.10)	26 (61.90)		
Rarely	89	14 (15.73)	75 (84.27)		
Sometimes	213	52 (24.41)	161 (75.59)		
Most of the time	184	15 (8.15)	169 (91.85)		
Always	79	15 (18.99)	64 (81.01)		
**Awkward posture**				38.66	< 0.001*
No	289	83 (28.72)	206 (71.28)		
Yes	318	29 (9.12)	289 (90.88)		
**Maintain good posture**				21.95	< 0.001*^a^
Never	28	11 (39.29)	17 (60.71)		
Rarely	104	7 (6.73)	97 (93.27)		
Sometimes	286	50 (17.48)	236 (82.52)		
Most of the time	168	41 (24.40)	127 (75.60)		
Always	21	3 (14.29)	18 (85.71)		

* Significant variable (p < 0.05)

^a^ p-values calculated from Fishers’ exact test.

### Organizational, behavioural and intervention factors influencing LBP

With respect to organizational factors, a significant association was revealed between understaffed (***χ*2** = 3.95, p = 0.047), and prevalence of LBP. Also, regarding intervention factors, trained on transport aids (***χ*2** = 4.05, p = 0.044) was found to be significantly related to LBP (Table 6).

**Table 6:** Organizational, behavioural and intervention factors influencing LBP.

Characteristics	*N*	LBP		*χ*2	p
		No	Yes		
**Understaffed**				3.95	0.047*
No	206	47 (22.82)	159 (77.18)		
Yes	401	65 (16.21)	336 (83.79)		
**Use of work procedures**				7.34	0.111^a^
Never	13	3 (23.08)	10 (76.92)		
Rarely	44	4 (9.09)	40 (90.91)		
Sometimes	95	25 (26.32)	70 (73.68)		
Most of the time	240	45 (18.75)	195 (81.25)		
Always	215	35 (16.28)	180 (83.72)		
**Trained on work machinery**				0.02	0.888
No	111	21 (18.92)	90 (81.08)		
Yes	496	91 (18.35)	405 (81.65)		
**Trained on transport aids**				4.05	0.044*
No	296	45 (15.20)	251 (84.80)		
Yes	311	67 (21.54)	244 (78.46)		
**Trained on good working posture**				0.58	0.446
No	225	38 (16.89)	187 (83.11)		
Yes	382	74 (19.37)	308 (80.63)		

* Significant variable (p < 0.05)

^a^ p-values calculated from Fishers’ exact test.

### Bivariate and multiple logistic regression of predisposing factors and prevalence of LBP

The Table 7 below summarizes the bivariate and multiple logistic regression analysis between predisposing factors and prevalence of LBP. In the bivariate logistic regression, factors such as age, type of employment, frequency of exercise, daily hours of sleep, working days in a typical week, overtime, on call duties, pressure from work, lifting heavy objects, transferring patients, awkward posture, maintain good posture, understaff, and trained on transport aids were associated with LBP at p < 0.05. However, on the multivariate logistic regression analysis, only age, daily hours of sleep, working days in a typical week, overtime, on call duties, prolong sitting, transferring patients, awkward posture, and understaff were related to LBP.

**Table 7:** Bivariate and multiple logistic regression of predisposing factors and prevalence of LBP.

Characteristics	Prevalence of LBP (n = 607)				
	*N*	COR (95% CI)	p	AOR (95% CI)	p
**Age**					
Median (IQR)	32.0 (28.0–37.0)	1.05 (1.02–1.09)	0.004*	1.07 (1.00–1.16)	0.044*
**Work experience**					
Median (IQR)	5.0 (3.0–12.0)	1.02 (0.99–1.06)	0.127	0.95 (0.88–1.03)	0.235
**Type of employment**					
Contract	95	1		1	
Permanent	512	2.40 (1.48–3.96)	< 0.001*	1.27 (0.61–2.62)	0.522
**Exercise frequently**					
No	299	1		1	
Yes	308	0.64 (0.42–0.97)	0.034*	0.81 (0.48–1.36)	0.426
**Daily hours of sleep**					
Less than 8	436	1		1	
8 and above	171	0.46 (0.30–0.71)	< 0.001*	0.54 (0.31–0.97)	0.038*
**Working days in a typical week**					
5 and below	493	1		1	
Above 5	114	7.70 (2.78–21.40)	< 0.001*	8.14 (2.65–25.02)	< 0.001*
**Overtime**					
No	297	1		1	
Yes	310	2.37 (1.55–3.65)	< 0.001*	2.00 (1.16–3.46)	0.013*
**On call duties**					
No	375	1		1	
Yes	232	1.60 (1.03–2.51)	0.036*	0.97 (0.54–1.74)	0.910
**Pressure from work**					
Not at all	28	1		1	
Occasionally	322	1.06 (0.43–2.60)	0.897	1.71 (0.58–5.08)	0.332
Frequently	257	2.73 (1.06–6.99)	0.037*	1.89 (0.60–5.97)	0.277
**Prolong sitting**					
Never	63	1		1	
Rarely	132	1.93 (0.81–4.61)	0.135	0.94 (0.33–2.71)	0.914
Sometimes	227	0.59 (0.29–1.20))	0.146	0.31 (0.12–0.80)	0.015*
Most of the time	141	1.03 (0.47–2.26)	0.939	0.80 (0.30–2.17)	0.671
Always	44	2.12 (0.63–7.14)	0.227	1.07 (0.26–4.51)	0.919
**Lifting heavy objects**					
Never	47	1		1	
Rarely	148	2.31 (1.11–4.82)	0.025*	1.54 (0.54–4.37)	0.421
Sometimes	266	2.07 (1.05–4.07)	0.034*	0.90 (0.31–2.65)	0.850
Most of the time	108	3.47 (1.52–7.90)	0.003*	0.69 (0.20–2.46)	0.569
Always	38	9.29 (1.98–43.62)	0.005*	3.55 (0.54–23.23)	0.186
**Transferring patients**					
Never	42	1		1	
Rarely	89	3.30 (1.42–7.67)	0.006*	3.22 (1.08–9.60)	0.035*
Sometimes	213	1.91 (0.95–3.82)	0.070	2.10 (0.76–5.82)	0.154
Most of the time	184	6.93 (3.06–15.69)	< 0.001*	6.95 (2.07–23.26)	0.002*
Always	79	2.63 (1.13–6.08)	0.024*	1.18 (0.36–3.92)	0.781
**Awkward posture**					
No	289	1		1	
Yes	318	4.02 (2.53–6.35)	< 0.001*	2.36 (1.31–4.25)	0.004*
**Maintain good posture**					
Never	28	1		1	
Rarely	104	8.97 (3.04–26.37)	< 0.001*	3.33 (0.87–15.21)	0.078
Sometimes	286	3.05 (1.35–6.92)	0.007*	1.40 (0.48–4.13)	0.541
Most of the time	168	2.00 (0.87–4.62)	0.103	1.49 (0.50–4.46)	0.475
Always	21	3.88 (0.92–16.36)	0.065	2.93 (0.51–17.04)	0.230
**Understaffed**					
No	206	1		1	
Yes	401	1.53 (1.00–2.33)	0.048*	1.84 (1.04–3.27)	0.036*
**Trained on transport aids**					
No	296	1		1	
Yes	311	0.65 (0.43–0.99)	0.045*	0.66 (0.39–1.11)	0.115

* Significant variable (p-value < 0.05); IQR–interquartile range; COR–crude odds ratio; AOR–adjusted odds ratio.

For every 1-year increase in age, the odds of experiencing LBP increase by 7% (AOR = 1.07, 95% CI = 1.00–1.16, p = 0.044). Working for more than 5 days in a typical week increased the odds of experiencing LBP by approximately 8 times (AOR = 8.14, 95% CI = 2.65–25.02, p < 0.001) compared to health workers who worked fewer than 5 days. Additionally, working for overtime increased the odds of experiencing LBP by 2 times (AOR = 2.00, 95% CI = 1.16–3.46, p = 0.013) than individuals who worked for no overtime. Again, health workers who were most of the time, and rarely involved in transferring patients increased their odds of experiencing LBP by closely 7 times (AOR = 6.95, 95% CI = 2.07–23.26, p = 0.002) and 3 times (AOR = 3.22, 95% CI = 1.08–9.60, p = 0.035), respectively, compared to those never engaged in the transfer of patients. Also, working in an awkward posture increased the odds of experiencing LBP by approximately 2 times (AOR = 2.36, 95% CI = 1.31–4.25, p = 0.004) than those who did not work in an awkward posture. Further, workers who are understaff in their department increased their odds of experiencing LBP by almost 2 times (AOR = 1.84, 95% CI = 1.04–3.27, p = 0.036).

However, getting a daily sleep duration of 8 hours or more reduced the odds of experiencing LBP by 46% (AOR = 0.54, 95% CI = 0.31–0.97, p = 0.038) in comparison with individuals who slept less than 8 hours. And the ability to engage in periods of sitting for some time during working hours decreased the odds of experiencing LBP by approximately 69% (AOR = 0.31, 95% CI = 0.12–0.80, p = 0.015) compared to individuals who never sat for long periods.

### Turnover intention related to the experience of LBP among health workers

The Table 8 depicts the turnover intention of study participants after their experience of LBP. Out of the 495 (81.6%) that experienced LBP, a substantial number, 123 (24.9%) and 144 (29.1%) occasionally consider leaving their job, and willing to accept another job at same compensation, respectively.

**Table 8:** Turnover intention related to experience of LBP among health workers.

Variable	*n*	Percentage (%)
**Considered leaving your job**		
Never	168	33.9
Rarely	102	20.6
Sometimes	123	24.9
Most of the times	59	11.9
Always	43	8.7
**Likely to accept another job at same compensation**		
Never	131	26.5
Rarely	107	21.6
Sometimes	144	29.1
Most of the times	62	12.5
Always	51	10.3

## Discussion

This study examined prevalence, predisposing factors and turnover intention related to LBP among health workers. More than three-quarters, 495 (81.9%), of healthcare workers experienced LBP in the past year. A notable proportion of lower back pain (LBP), 175 (35.4%), persisted for longer than three months and were classified as chronic. Advanced age, working more than five days per week, and overtime work were linked to higher odds of experiencing LBP. Additionally, involvement in patient transfers, working in awkward postures, and being understaffed were also associated with increased odds of experiencing LBP. Nevertheless, getting 8 hours or more of sleep per day and sitting intermittently during work were linked to reduced odds of experiencing LBP. Among the health workers who reported experiencing LBP, a significant number, 24.9%, occasionally contemplates leaving their current job, while 29.1% express willingness to accept another job with similar compensation.

In this present study, LBP prevalence of 81.6% was reported in the past year. This was parallel to studies conducted in Nigeria (87.3%) [39], South Africa (79.3) [40], Saudi Arabia (81.4%) [41] and China (80.0%) [42]. This similarity might be due to operational definition of LBP, study design, work settings and healthcare delivery system. Nonetheless, the proportion of LBP in this study was lower than a recent study conducted in Serbia (94.0%) [43] but higher than those conducted in Uganda (39.6%) [30] and Ethiopia (57.5%) [44]. This inconsistency may be due to socio-demographic characteristics of study participants, discrepancies in pain reporting culture and lifestyle. For instance, in Asian cultures, there is a cultural norm of anticipating a dramatic expression of emotion when confronted with pain, whereas in African societies, there is a preference for resilience, self-control, and minimizing the display of pain [45,46]. Furthermore, African individuals may perceive mild pain as a usual occurrence and might not readily report it as being in pain [47]. On the contrary, the proportion of LBP in this study was higher than a study carried out in Brazil (65.2%) [48], Uganda (39.6%) [30], Ethiopia (57.5%) [49] and Nigeria (67.6%) [50]. Also, some studies conducted in Ghana recorded a prevalence of 73.3% [51], 51.2% [25] and 49.5% [26], which were lower than this present study. These variations may be due to different work settings, workload and the number of health facilities involved in a study. For instance, the previous studies in Ghana were conducted in one or two facilities in a region, and the workload of workers in those regions may be less and may have contributed to the low prevalence of LBP.

This current study found that an increase in age was associated with higher odds of experiencing LBP. This finding was similar to studies conducted in different parts of the world [6,12,18,44,52–55]. As health workers age, the risk of experiencing LBP increases due to degenerative changes in spinal discs, muscle weakness, reduced flexibility, slower recovery, and potential development of chronic conditions [56]. Also, as health workers advance in their careers, they may take on roles with higher levels of responsibility, which can include supervising and training junior staff, making critical decisions, and managing complex medical cases. These demanding roles may expose and put them at higher rate of experiencing LBP.

In this study, health workers who worked for 5 days and above had a higher odd of experiencing LBP compared to those who worked for less than 5 days. This outcome is in agreement with studies carried in Poland and the United States, where workers who worked for more than 40 hours a week were significantly at a higher risk of exposure to LBP [57,58]. This association could be attributed to factors like prolonged sitting or standing, repetitive movements, heavy lifting, inadequate rest breaks, poor ergonomics, and high levels of stress. Consequently, these factors can put strain on the lower back, leading to discomfort and pain [59]. Working more days without adequate breaks can cause persistent muscle fatigue, weakening the body’s ability to recover from daily strain. Over time, this can lead to chronic lower back pain and discomfort due to decreased muscle strength and reduced joint mobility [60].

Additionally, health workers who worked overtime increased their odds of experiencing LBP in the current study. This finding is similar to a study conducted in Bangladesh and China [61,62]. Research shows that workers who work overtime or exceed eight hours a day are more likely to experience low back pain. A study revealed that these workers have almost double the risk of developing low back pain compared to those working standard hours [63]. The increased pain is linked to prolonged exposure to physical stressors, such as repetitive movements and poor body mechanics, commonly found in industrial environments. Overtime often involves prolonged manual handling tasks without adequate rest periods, resulting in repetitive strain. This strain leads to muscle fatigue and injury, especially in the lower back [64]. Prolonged exposure to heavy lifting and awkward postures over extended periods increases the risk of musculoskeletal disorders including low back pain. Health workers in most places may need to work overtime due to inadequate staff. This prolongs their already demanding work at the hospital and may lead to frequent development of LBP.

Besides, in this current investigation, workers in health facilities with inadequate staff were at a higher risk of experiencing LBP. The results of studies conducted by Kim et al. [65] and Sanjoy et al. [62] in the United States of America and Bangladesh, respectively, supported this finding. Also, insufficient clinical and supporting staff in a hospital could increase the number of manual handling tasks per worker, along with overtime work, consequently elevating the likelihood of experiencing LBP [62]. The issue of understaff is common to many healthcare settings, and it’s not surprising that studies conducted in different settings had comparable results. Additionally, in a healthcare facility that is understaffed, individual staff members may have to handle a higher workload, including lifting and moving patients, without sufficient support or assistance [19]. This increased demand can lead to more frequent and prolonged manual handling tasks, which can put a strain on the lower back muscles and spine.

In this study, health workers who were involved in transferring patient had a greater odd of experiencing LBP than those who never transferred patient in their line of work. Similar studies carried out in Saudi Arabia [66] and Nigeria [12,50] have confirmed this finding. Health workers involved in transferring of patients often must lift, move, or reposition them, which can put immense strain on their lower back muscles and spine. Repeatedly performing these physical tasks, especially with improper body mechanics, can lead to musculoskeletal disorders, including LBP [67].

Again, according to the findings of this recent study, working in awkward posture increases the odds of experiencing LBP. This outcome of the research was comparable to the ones conducted among Ugandan and Ethiopian health workers [30,68]. These similarities in results may be due to similar ways of health workers in these African countries adopt unnatural or uncomfortable positions while sitting, standing, or lifting during work, which puts additional stress on the muscles, ligaments, and discs in the lower back. Moreover, prolonged periods of maintaining awkward postures can lead to muscle fatigue, strain, and increased pressure on the spine, increasing the likelihood of developing LBP.

Nonetheless, this current study also found that sleeping for a duration of 8 hours and above reduces the odds of experiencing LBP. This evidence is consistent with a study conducted in China, where it was found that longer sleeping hours decreased the experience of LBP [69,70]. The insufficient duration of sleep may affect all health workers in the globe and can significantly influences the chance of experiencing LBP. Insufficient or poor-quality sleep can hinder muscle recovery, increase pain sensitivity, impair cognitive function, and disrupt inflammation regulation [71]. Prioritizing adequate sleep and practicing good sleep hygiene can help reduce the risk of LBP and promote overall well-being.

In this study, health workers who had intermittent rest periods during work hours had reduced odds of experiencing LBP. This outcome is consistent with studies conducted in Turkey, Ethiopia and Norway [44,52,72]. Taking breaks or sitting intermittently during working hours help reduce muscle fatigue, improve circulation, maintain spinal alignment, decrease spinal compression, and promote movement, contributing to reduction of exposure to LBP, and better overall musculoskeletal health [73].

Though a couple of studies have predicted a strong association between LBP and long-term sickness absence [15–17,74], and motivation and job satisfaction on turnover intention [75], there is lack of studies that examined turnover intention after experiencing LBP among health workers. However, in this current study, a substantial number of study participants occasionally considered leaving their job and were willing to accept another job at same compensation, respectively, after experiencing LBP. Therefore, further studies are necessary to examine the effect of LBP on turnover intention in the midst of the recent rising number of health workers leaving the health industry, both in Ghana and the globe [75,76].

The findings of this study have significant implications for policy and practice in the healthcare sector. Policymakers and healthcare administrators should prioritize strategies to mitigate the risk of lower back pain (LBP) among healthcare workers, such as implementing ergonomic interventions, including enhanced ergonomic training and application through regular reminders, visual cues, and periodic "posture refreshers". Also, ensuring access to assistive lifting devices, such as mechanical lifts, and encouraging a culture that prioritizes employee health and safety by regularly addressing occupational health risks are also crucial. Additionally, establishing a reporting system for staff to communicate ergonomic concerns or report instances of musculoskeletal pain can facilitate prompt interventions. Further, policies limiting overtime work, ensuring adequate staffing levels, promoting healthy sleep habits, and encouraging regular breaks during work hours can help reduce the prevalence of LBP. Furthermore, healthcare institutions should also provide training on proper lifting techniques, patient transfer protocols, and stress management to minimize the risk of LBP. By addressing these modifiable risk factors, healthcare organizations can reduce the burden of LBP, improve worker well-being, and maintain high-quality patient care.

### Strength and limitations

The research took place among health workers chosen from 10 major facilities in the National Capital of Ghana, aiming to mirror the overall situation in the country. However, there are certain constraints associated with this study. The utilization of a cross-sectional study approach means that it cannot establish definitive cause-and-effect relationships or ascertain the sequence of causation between different factors. Furthermore, the study is susceptible to recall bias, as participants were questioned about events from the previous 12 months. Also, the generalization of the study findings may be applicable to only major health facilities due to sampling of participants from only major hospitals.

## Conclusion

The prevalence of LBP among health workers in the Greater Accra region was high. Advanced age, working more than 5 days in a typical work week, working overtime, being involved in transferring patients, working in awkward posture, understaffed were significant associated with higher risk of exposure to LBP. On the contrary, sleeping for 8 hours or more, and ability to sit regularly during work were related to lower likelihood of experiencing LBP. A concerned proportion indicated a strong intention to leave their current job position due to experiences of LBP. Health administrators and policy makers should consider ways of addressing these occupational, organizational and demographic factors to reduce the exposure of health workers to LBP. Future research can utilize prospective cohort studies as research evidence in order to establish causal relationships, and further investigate the effect of LBP on turnover intention.

## Supporting information

S1 File The raw data supporting findings of the article. (PDF)

S2 File Data collection tool for the study. (XLSX)

## References

[1] Cunningham C, Flynn T, Blake C. Low back pain and occupation among Irish health service workers. Occupational Medicine. 2006;56(7):447–54. doi: 10.1093/occmed/kql056 16793854

[2] Sharma S, Traeger AC, Mishra SR, Sharma S, Maher CG. Delivering the right care to people with low back pain in low-and middle-income countries: the case of Nepal. Journal of global health. 2019;9(1). doi: 10.7189/jogh.09.010304 30774940 PMC6367652

[3] Shokri P, Zahmatyar M, Falah Tafti M, Fathy M, Rezaei Tolzali M, Ghaffari Jolfayi A, et al. Non‐spinal low back pain: Global epidemiology, trends, and risk factors. Health Science Reports. 2023 Sep;6(9):e1533. doi: 10.1002/hsr2.1533 37674621 PMC10477419

[4] Kamper SJ, Apeldoorn AT, Chiarotto A, Smeets RJEM, Ostelo RWJG, Guzman J, et al. Multidisciplinary biopsychosocial rehabilitation for chronic low back pain: Cochrane systematic review and meta-analysis. BMJ. 2015 Feb 18;350(feb18 5):h444–h444. doi: 10.1136/bmj.h444 25694111 PMC4353283

[5] Vos T, Flaxman AD, Naghavi M, Lozano R, Michaud C, Ezzati M, et al. Years lived with disability (YLDs) for 1160 sequelae of 289 diseases and injuries 1990–2010: a systematic analysis for the Global Burden of Disease Study 2010. The lancet. 2012;380(9859):2163–96. doi: 10.1016/S0140-6736(12)61729-2 23245607 PMC6350784

[6] Wang DY, Sun YY. Prevalence and influencing factors of low back pain among nurses in China: a systematic review and meta-analysis. Frontiers of Nursing. 2020 Dec 1;7(4):329–36.

[7] Manchikanti L, Singh V, Falco FJ, Benyamin RM, Hirsch JA. Epidemiology of low back pain in adults. Neuromodulation: Technology at the Neural Interface. 2014;17:3–10. 10.1111/ner.12018 25395111

[8] Landry MD, Raman SR, Sulway C, Golightly YM, Hamdan E. Prevalence and risk factors associated with low back pain among health care providers in a Kuwait hospital. Spine. 2008;33(5):539–45. doi: 10.1097/BRS.0b013e3181657df7 18317200

[9] Lipscomb J, Trinkoff A, Brady B, Geiger-Brown J. Health care system changes and reported musculoskeletal disorders among registered nurses. American journal of public health. 2004;94(8):1431–5. doi: 10.2105/ajph.94.8.1431 15284055 PMC1448467

[10] Bejia I, Younes M, Jamila HB, Khalfallah T, Salem KB, Touzi M, et al. Prevalence and factors associated to low back pain among hospital staff. Joint bone spine. 2005;72(3):254–9. doi: 10.1016/j.jbspin.2004.06.001 15850998

[11] Wong T, Teo N, Kyaw M. Prevalence and risk factors associated with low back among health care providers in a District Hospital. Malaysian Orthopaedic Journal. 2010;4(2):23–8.

[12] Awosan KJ, Yikawe SS, Oche OM, Oboirien M. Prevalence, perception and correlates of low back pain among healthcare workers in tertiary health institutions in Sokoto, Nigeria. Ghana Med J. 2017 Dec;51(4):164–74. doi: 10.1016/s0140-6736 29622830 PMC5870785

[13] Vieira ER, Kumar S, Coury HJ, Narayan Y. Low back problems and possible improvements in nursing jobs. Journal of advanced nursing. 2006;55(1):79–89. doi: 10.1111/j.1365-2648.2006.03877.x 16768742

[14] Sikiru L, Hanifa S. Prevalence and risk factors of low back pain among nurses in a typical Nigerian hospital. African health sciences. 2010;10(1):26–30. 20811521 PMC2895788

[15] Andersen LL, Burdorf A, Fallentin N, Persson R, Jakobsen MD, Mortensen OS, et al. Patient transfers and assistive devices: prospective cohort study on the risk for occupational back injury among healthcare workers. Scand J Work Environ Health. 2014 Jan;40(1):74–81. doi: 10.5271/sjweh.3382 24030699

[16] Holtermann A, Clausen T, Aust B, Mortensen OS, Andersen LL. Risk for low back pain from different frequencies, load mass and trunk postures of lifting and carrying among female healthcare workers. Int Arch Occup Environ Health. 2013 May;86(4):463–70. doi: 10.1007/s00420-012-0781-5 22585061

[17] Wang SY, Liu LC, Lu MC, Koo M. Comparisons of Musculoskeletal Disorders among Ten Different Medical Professions in Taiwan: A Nationwide, Population-Based Study. Dalal K, editor. PLoS ONE. 2015 Apr 10;10(4):e0123750. doi: 10.1371/journal.pone.0123750 25861017 PMC4393309

[18] Rezaei B, Mousavi E, Heshmati B, Asadi S. Low back pain and its related risk factors in health care providers at hospitals: A systematic review. Ann Med Surg (Lond). 2021 Oct;70:102903. doi: 10.1016/j.amsu.2021.102903 34691437 PMC8519806

[19] Kasa AS, Workineh Y, Ayalew E, Temesgen WA. Low back pain among nurses working in clinical settings of Africa: systematic review and meta-analysis of 19 years of studies. BMC Musculoskelet Disord. 2020 May 16;21(1):310. 32416726 10.1186/s12891-020-03341-y PMC7231416

[20] Boateng AB, Opoku DA, Ayisi-Boateng NK, Sulemana A, Mohammed A, Osarfo J, et al. Factors Influencing Turnover Intention among Nurses and Midwives in Ghana. Suksatan W, editor. Nursing Research and Practice. 2022 Nov 16;2022:1–8. 10.1155/2022/4299702 PMC9683982 36439941

[21] Boateng R, Boateng AA, Department of Health Administration and Education, University of Education, Winneba, Ghana, Aboagye G, Kumah E, Department of Health Administration and Education, University of Education, Winneba, Ghana, et al. Training Motivation and Post Training Turnover Intention: Reifying the Narrative in a Community Health Nursing Training Institution in Ghana. J Econ Managem Res. 2023 May 31;1–5.

[22] Konlan KD, Asampong E, Dako-Gyeke P, Glozah FN. Burnout syndrome among healthcare workers during COVID-19 Pandemic in Accra, Ghana. Mortazavi F, editor. PLoS ONE. 2022 Jun 16;17(6):e0268404. doi: 10.1371/journal.pone.0268404 35709139 PMC9202923

[23] Bonenberger M. Can health workforce management actions positively influence retention and attrition of health workers?: a study on human resources for health in the eastern region of Ghana. 2015 [cited 2024 Apr 5]; Available from: https://edoc.unibas.ch/39161/

[24] Tawiah PA, Baffour-Awuah A, Effah ES, Adu-Fosu G, Ashinyo ME, Alhassan RK, et al. Occupational health hazards among healthcare providers and ancillary staff in Ghana: a scoping review. BMJ Open. 2022 Oct;12(10):e064499. doi: 10.1136/bmjopen-2022-064499 36283753 PMC9606738

[25] Nyame-Annan EKP. Occupational Hazards and Safety Practices among Hospital Workers at Greater Accra Regional Hospital, Ridge. 2017.

[26] Awudu L. Occupational health and safety practices among healthcare workers in some selected hospitals in tamale metropolis. [Tamale, Ghana]: University of Development Studies; 2018.

[27] University of Ghana School of Public Health. State of the Nation’s Health Report. Ghana: University of Ghana, School of Public Health; 2018 Dec p. 164.

[28] Ghana Statistical Service. Ghana 2021 Population and Housing Census General Report Volume 3A. Accra: Ghana Statistical Service; 2021 p. 1–112.

[29] Cochran WG. Sampling Techniques. 3rd Edition. New York: John Wiley & Sons, Inc.; 1977.

[30] Aleku M, Nelson K, Abio A, Lowery Wilson M, Lule H. Lower Back Pain as an Occupational Hazard Among Ugandan Health Workers. Front Public Health. 2021 Dec 1;9:761765. doi: 10.3389/fpubh.2021.761765 34926384 PMC8671744

[31] Kaiser R, Woodru BA, Bilukha O, Spiegel PB, Salama P. Using design effects from previous cluster surveys to guide sample size calculation in emergency settings. Disaster. 2006;30(2):199–211. doi: 10.1111/j.0361-3666.2006.00315.x 16689918

[32] Rowe AK, Lama M, Onikpo F, Deming MS. Design effects and intraclass correlation coefficients from a health facility cluster survey in Benin. International Journal for Quality in Health Care. 2002 Dec 1;14(6):521–3. doi: 10.1093/intqhc/14.6.521 12515339

[33] Neyman J. One the two different aspects of representative method: The method of Stratified Sampling and the method of purposive selection. J Roy Satist Soc. 1934;97(4):558–606.

[34] Neyman J. Contribution to the theory of sampling human populations. Journal of the American Statistical Association. 1938;33(201):101–16.

[35] Nyonator F, Ofosu A, Osei D. District Health Information Management System DHIMS II: The Data Challenge For Ghana Health Service. Accra: Policy Planning Monitoring and Evaluation Division, Ghana Health Service. 2013;

[36] Centre for Disease Control and Prevention, The National Institute for Occupational Safety and Health. Healthcare Workers Safety and Health [Internet]. 2021 [cited 2022 May 3]. Available from: https://www.cdc.gov/niosh/docket/archive/docket135.html

[37] Hartung C, Lerer A, Anokwa Y, Tseng C, Brunette W, Borriello G. Open data kit: tools to build information services for developing regions. In: Proceedings of the 4th ACM/IEEE International Conference on Information and Communication Technologies and Development—ICTD ‘10 [Internet]. London, United Kingdom: ACM Press; 2010 [cited 2022 May 6]. p. 1–12. Available from: http://dl.acm.org/citation.cfm?doid=2369220.2369236

[38] StataCorp. Stata Statistical Software: Release 15. College Station, TX: StataCorp LLC; 2017.

[39] Olatubi MI, Alabi BD, Ademuyiwa GO, Ojo IO. Prevalence and Management of Low Back Pain Among Health Workers in a Privately Owned Teaching Hospital in Nigeria. TOPHJ. 2022 Dec 30;15(1):e187494452211230.

[40] Khumalo K, Haffejee F. Prevalence and associated risk factors of low back pain among users of a primary health care clinic serving semi-urban and rural settlements in KwaZulu-Natal, South Africa. Afr H Sci. 2022 Aug 1;22(2):592–601. doi: 10.4314/ahs.v22i2.68 36407349 PMC9652636

[41] Al Amer HS. Low back pain prevalence and risk factors among health workers in Saudi Arabia: A systematic review and meta-analysis. J Occup Health. 2020 Jan;62(1):e12155. doi: 10.1002/1348-9585.12155 32710807 PMC7382437

[42] Yang S, Lu J, Zeng J, Wang L, Li Y. Prevalence and Risk Factors of Work-Related Musculoskeletal Disorders Among Intensive Care Unit Nurses in China. Workplace Health Saf. 2019 Jun;67(6):275–87. doi: 10.1177/2165079918809107 30582426

[43] Bozic A, Gajdobranski D, Brestovacki-Svitlica B, Medic-Pericevic S, Mikov M, Vasovic V, et al. The prevalence of low back pain among nurses in Serbia. Work. 2022;71(1):249–54. doi: 10.3233/WOR-205144 34924418

[44] Negash NA, Tadele A, Jember Ferede A. Prevalence and Associated Factors of Low Back Pain Among Healthcare Professionals at University of Gondar Comprehensive and Specialized Hospital, Northwest Ethiopia: Cross-Sectional Study. JPR. 2022 May;Volume 15:1543–52. doi: 10.2147/JPR.S351987 35642186 PMC9148573

[45] Ehrhardt MD, Gray KN, Kuhn BL, Lannon EW, Palit S, Sturycz CA, et al. A qualitative analysis of pain meaning: results from the Oklahoma Study of Native American Pain Risk (OK-SNAP). Ethn Health. 2020;27(3):721–32. doi: 10.1080/13557858.2020.1760215 32378419

[46] Henschke N, Lorenz E, Pokora R, Michaleff ZA, Quartey JNA, Oliveira VC. Understanding cultural influences on back pain and back pain research. Best Pract Res Clin Rheumatol. 2016 Dec;30(6):1037–49. doi: 10.1016/j.berh.2017.08.004 29103548

[47] Tefera BZ, Zeleke H, Abate A, Abebe H, Mekonnen Z, Sewale Y. Magnitude and associated factors of low back pain among nurses working at intensive care unit of public hospitals in Amhara region, Ethiopia. Abdullah KL, editor. PLoS ONE. 2021 Dec 2;16(12):e0260361. doi: 10.1371/journal.pone.0260361 34855797 PMC8639077

[48] Corrêa Pinto RN, da Silva MC, Caputo EL, Domingues MR. Low back pain prevalence and associated factors in nurses from Brazilian primary health units. Work. 2021;70(1):279–85. doi: 10.3233/WOR-213572 34511471

[49] Gashawbeza B, Ezo E. Prevalence and factors associated with low back pain among health care providers in public hospitals of Gamo zone, Southern Ethiopia. SAGE Open Medicine. 2022 Jan;10:205031212211143. 10.1177/20503121221114311 PMC9310333 35898955

[50] Ajayi P, Deji S, Adewoye K, Atoyebi O, Alabi A, Solomon O, et al. Determinants of low back pain among health-care providers in a federal tertiary hospital in Ekiti State, Southwestern Nigeria. Niger J Med. 2021;30(4):374.

[51] Abla Kofi- Bediako W, Sama G, Yarfi C, Ed-Bansah D, Appah Acquah A. Work-Related Musculoskeletal Disorders among Nurses at the Ho Teaching Hospital, Ghana. Proceedings of the Human Factors and Ergonomics Society Annual Meeting. 2021 Sep;65(1):1291–4. 10.1177/1071181321651256 PMC8809478 35115743

[52] Şimşek Ş, Yağcı N, Şenol H. Prevalence of and risk factors for low back pain among healthcare workers in Denizli. Agri. 2017 Apr;29(2):71–8. doi: 10.5505/agri.2017.32549 28895982

[53] Nottidge TE, Nottidge BA, Ekrikpo UE. Prevalence and predictors of low back pain in a Southern Nigerian hospital. Ann Afr Med. 2019 Sep;18(3):167–72. doi: 10.4103/aam.aam_59_18 31417018 PMC6704812

[54] Latina R, Petruzzo A, Vignally P, Cattaruzza MS, Vetri Buratti C, Mitello L, et al. The prevalence of musculoskeletal disorders and low back pain among Italian nurses: An observational study. Acta Biomed. 2020 Nov 30;91(12-S):e2020003. doi: 10.23750/abm.v91i12-S.10306 33263343 PMC8023105

[55] Brusini A. Low back pain among nurses in Italy: a review. G Ital Med Lav Ergon. 2021 Dec;43(4):369–72. 35049161

[56] Lela M, Frantz JM. Physical activity among nurses in Kanombe Military Hospital. African Journal of Physiotherapy and Rehabilitation Sciences. 2012;4(1–2):63–6.

[57] Mroczek B, Lubkowska W, Jarno W, Jaraczewska E, Mierzecki A. Occurrence and impact of back pain on the quality of life of healthcare workers. Annals of Agricultural and Environmental Medicine. 2020;27(1):36–42. doi: 10.26444/aaem/115180 32208577

[58] Yang H, Haldeman S, Lu ML, Baker D. Low back pain prevalence and related workplace psychosocial risk factors: a study using data from the 2010 National Health Interview Survey. Journal of manipulative and physiological therapeutics. 2016;39(7):459–72. doi: 10.1016/j.jmpt.2016.07.004 27568831 PMC5530370

[59] El-Soud AMA, El-Najjar AR, El-Fattah NA, Hassan AA. Prevalence of low back pain in working nurses in Zagazig University Hospitals: an epidemiological study. Egypt Rheumatol Rehabil. 2014 Jul;41(3):109–15.

[60] Kasumawati F, Adha MZ, Azizah FN, Ramuni K, Katta R. Correlation between length of work and work posture with low back pain complaint among back office employees at X Hospital Serpong District, South Tangerang, Indonesia. South Tangerang, Indonesia. 2020;16(8):34–7.

[61] Lin PH, Tsai YA, Chen WC, Huang SF. Prevalence, characteristics, and work-related risk factors of low back pain among hospital nurses in Taiwan: A cross-sectional survey. International Journal of Occupational Medicine and Environmental Health [Internet]. 2012 Jan 1 [cited 2023 Jul 24];25(1). Available from: http://ijomeh.eu/Prevalence-characteristics-and-work-related-risk-factors-of-low-back-pain-among-hospital-nurses-in-taiwan-a-cross-sectional-survey,2278,0,2.html doi: 10.2478/s13382-012-0008-8 22219056

[62] Sanjoy SS, Ahsan GU, Nabi H, Joy ZF, Hossain A. Occupational factors and low back pain: a cross-sectional study of Bangladeshi female nurses. BMC Res Notes. 2017 Dec;10(1):173. doi: 10.1186/s13104-017-2492-1 28454550 PMC5410056

[63] Chowdhury MdOSA, Huda N, Alam MdM, Hossain SI, Hossain S, Islam S, et al. Work-related risk factors and the prevalence of low back pain among low-income industrial workers in Bangladesh: results from a cross-sectional study. Bull Fac Phys Ther. 2023 Jun 7;28(1):20.

[64] Sambeko BEM, Susanto N, Alfanan A. Manual Handling as Contributor of Low Back Pain for Workers: A Case Study at PT Sumber Mandiri Jaya, Kabupaten Merauke. IJOSH. 2023 Mar 31;13(1):29–36.

[65] Kim SS, Okechukwu CA, Dennerlein JT, Boden LI, Hopcia K, Hashimoto DM, et al. Association between perceived inadequate staffing and musculoskeletal pain among hospital patient care workers. Int Arch Occup Environ Health. 2014 Apr;87(3):323–30. doi: 10.1007/s00420-013-0864-y 23475312 PMC5321209

[66] Almaghrabi A, Alsharif F. Prevalence of Low Back Pain and Associated Risk Factors among Nurses at King Abdulaziz University Hospital. Int J Environ Res Public Health. 2021 Feb 7;18(4):1567. doi: 10.3390/ijerph18041567 33562299 PMC7914573

[67] Tinubu BM, Mbada CE, Oyeyemi AL, Fabunmi AA. Work-Related Musculoskeletal Disorders among Nurses in Ibadan, South-west Nigeria: a cross-sectional survey. BMC Musculoskelet Disord. 2010 Dec;11(1):12. doi: 10.1186/1471-2474-11-12 20089139 PMC2823665

[68] Mijena GF, Geda B, Dheresa M, Fage SG. Low back pain among nurses working at public hospitals in eastern Ethiopia. Journal of pain research. 2020;1349–57. doi: 10.2147/JPR.S255254 32606901 PMC7292259

[69] Niu J, An Y, Xu M, Zhang L, Liu J, Feng X, et al. Do sleep and psychological factors influence musculoskeletal pain among nurses? Work. 2023;(Preprint):1–11. doi: 10.3233/WOR-211113 36710694

[70] Wang M, Yu J, Liu N, Liu Z, Wei X, Yan F, et al. Low back pain among taxi drivers: a cross-sectional study. Occupational Medicine. 2017 Jun;67(4):290–5. doi: 10.1093/occmed/kqx041 28498976

[71] Sutton BC, Opp MR. Musculoskeletal sensitization and sleep: chronic muscle pain fragments sleep of mice without altering its duration. Sleep. 2014;37(3):505–13. doi: 10.5665/sleep.3486 24587573 PMC3920316

[72] Lunde LK, Koch M, Knardahl S, Veiersted KB. Associations of objectively measured sitting and standing with low-back pain intensity: a 6-month follow-up of construction and healthcare workers. Scandinavian journal of work, environment & health. 2017;269–78. doi: 10.5271/sjweh.3628 28272649

[73] Takala EP. Ergonomic interventions and prevention–a need for better understanding of implementation. Scandinavian Journal of Work, Environment & Health. 2018;44(2):111–2. doi: 10.5271/sjweh.3710 29355290

[74] Andersen LL, Clausen T, Mortensen OS, Burr H, Holtermann A. A prospective cohort study on musculoskeletal risk factors for long-term sickness absence among healthcare workers in eldercare. Int Arch Occup Environ Health. 2012 Aug;85(6):615–22. doi: 10.1007/s00420-011-0709-5 21986907

[75] Bonenberger M, Aikins M, Akweongo P, Wyss K. The effects of health worker motivation and job satisfaction on turnover intention in Ghana: a cross-sectional study. Hum Resour Health. 2014 Dec;12(1):43. doi: 10.1186/1478-4491-12-43 25106497 PMC4130118

[76] Poon YSR, Lin YP, Griffiths P, Yong KK, Seah B, Liaw SY. A global overview of healthcare workers’ turnover intention amid COVID-19 pandemic: a systematic review with future directions. Hum Resour Health. 2022 Sep 24;20(1):70. doi: 10.1186/s12960-022-00764-7 36153534 PMC9509627

