# Supplementary material for: Prevalence, predisposing factors, and turnover intention related to low back pain among health workers in Accra, Ghana
Source: PLoS One. 2025 Jan 16;20(1):e0317582. doi: 10.1371/journal.pone.0317582 (PMC11737759; doi:10.1371/journal.pone.0317582)
Supplement: Attachment — Submitted filename: Report on Manuscript - Back pain on turnover intention - 06.11.23.pdf [file pone.0317582.s003.pdf]

Report on Manuscript: PONE-D-23-26548: Predisposing factors and turnover intention effects of low back pain among health workers in selected hospitals in the Greater Accra region, Ghana

I have read the above manuscript and have made comments on some of the key issues that require the attention of the author(s).

A. Structure of manuscript
The author(s) structured the manuscripts as below.

Introduction

Materials and Methods
Study design, participants and setting
Sample size determination
Sampling procedure
Inclusion and exclusion criteria
Data collection
Data management and analysis
Ethical consideration

Results
Socio-demographic and lifestyle characteristics of Health Workers
Occupational related factors
Organizational, behavioural and intervention related factors
Prevalence of exposure to Low back pain among Health Workers
Socio-demographic and lifestyle characteristics influencing Low back pain
Occupational factors influencing Low back pain Exposure
Organizational, behavioural and intervention factors influencing low back pain exposure
Bivariate and multiple logistic regression of risk factors and exposure to low back pain
Turnover Intention Effects of Low back pain on Health Workers

Discussion
Strength and limitations

Conclusion

B. Comments and suggestions
I provide the following suggestions on the manuscript.

Objective(s) of the study
The author(s) indicated in the abstract that 'This study investigated the prevalence, predisposing factors, and turnover intention effects of LBP among health workers in Ghana.' (page 2).

The author(s) stated in the introduction section that 'This study, therefore, investigated the prevalence, predisposing factors and turnover intention effects of low back pain among healthcare providers and ancillary staff in the Greater Accra region of Ghana' (page 4).

The author(s) should ensure consistency in the statement of the objectives in the abstract, and the introduction section. More importantly, deducing from the two objectives stated above, there is a lack of clarity on the focus of the study – what is the dependent or outcome variable of the study?

There is also a lack of coherence between the title of the paper and the objective of the study. The title of the paper is 'Predisposing factors and turnover intention effects of low back pain among health workers in selected hospitals in the Greater Accra region, Ghana'

This account for the difficult in devising an objective to reflect what the study sought to achieve. Perhaps, the above challenge(s) could be addressed if the title is rephrased. For instance, the author(s) could consider the following;

1. 'Effects of low back pain on turnover intention among health workers in selected hospitals in the Greater Accra Region, Ghana'

2. 'Factors associated with low back pain and its effect on turnover intention among health workers in selected hospitals in the Greater Accra Region, Ghana'

The objectives related to the above suggested titles could be stated as:

1. 'To determine the effects of low back pain on turnover intention among health workers in selected hospitals in the Greater Accra Region, Ghana'

2. 'To assess factors associated with low back pain and its effect on turnover intention among health workers in selected hospitals in the Greater Accra Region, Ghana'

For the purpose of the comments in this report, the second title and second objective stated above would be considered. This will mean that the dependent variable or outcome will be turnover intention. In this case, low back pain could be considered as the intermediate variable to turnover intention.

Restructuring the manuscript

Unless it is the requirement of the journal, the author(s) could restructure the manuscript as below to ensure that the reader is able to follow the line of argument with ease.

Introduction

The author(s) presented a section titled above where there was an analysis of extant literature on the topic under discussion (pages 3-4). Perhaps, a separation of the introduction from the literature review and theoretical perspective would make it easy for the reader as well. Thus, the author(s) should seek to present an analysis of the problem, limitations in the extant literature and provide the justification for the current study (as partly done) in the introduction section here. The research objective should follow from the above as stated in the concluding paragraph of the section (this should take account of the needed revision to be made).

Literature review

There should be a section as above and be re-arranged to let the reader know what the literature review part is, and what the theoretical perspective part is, as suggested below.

Literature review of related studies

There should be a part of the section that explains to the reader what has been written about the topic and the concepts therein. For instance, there should be a clearer analysis of studies related to prevalence/proportion of low back pain among health workers, and prevention/proportion of turnover intention among health workers. There should also be an analysis of studies related to how predisposing (socio-demographic characteristics) factors, organisational factors, lifestyle factors, behavioural factors and intervention factors have been associated with low back pain and turnover intention among health workers. These concepts should come out clearly (even if presented in paragraphs) in the write up.

Theoretical perspective

Even though the author(s) indicated that there were predisposing factors associated with low back pain and turnover intention (emphasis mine), there was no theoretical foundation stated. Perhaps, the Andersen (1995) healthcare utilisation model which projects predisposing factors, enabling factors and need factors could be considered. This could help to show a linkage between the socio-demographic characteristics as the predisposing factors, the organisational factors as enabling factors and lifestyle/behavioural factors as the need factors in a conceptual framework. The author(s) should ensure that the tenets of the chosen theory/theories are properly explained and supported by studies that have applied them before (even if presented in paragraphs) in the write up.

Methods

The author(s) presented a section titled 'Materials and Methods' with subsections (pages 4-8). The author(s) should seek to revise the section to portray the following key subsections.

Study Design

The author(s) presented a subsection titled 'Study design, participants and setting' and stated that 'An analytic facility-based cross sectional study design with a quantitative approach was employed for this research' (page 4). This section should focus on the study design as titled above. The author(s) should present a theoretical definition a justification for the choice of this study design as well as the quantitative research method applied.

Study Area

The author(s) presented the study area as part of a subsection titled 'Study design, participants and setting' and stated that 'The study was carried … ten (10) hospitals in the Greater Accra region of Ghana… The Greater Accra region is one of the sixteen regions of Ghana (22) … The region is regarded as an area with the highest population density due to its high in-migration and population growth rate (24)' (pages 4-5).

This description failed to provide the international reader with a clearer idea of the socio-economic challenges impeding access to healthcare among people in the study area. The author(s) should seek to explain to the reader further background of the Greater Accra Region and Ghana in which these hospitals are located in terms of geography, demography, economic status - before the description of health care provision (in sequence/paragraphs). This analysis will enable the international reader to understand the setting of the study and the justification for the choice based on these backgrounds. These should precede the description of the selected hospitals.

Study population
The author(s) presented the study population as part of a subsection titled 'Study design, participants and setting' and stated that 'The study was carried out among physicians, nurses, midwives, medical laboratory staff, physiotherapists, health care assistants, orderlies and laundry workers of ten (10) hospitals in the Greater Accra region of Ghana' (page 4). The author(s) should provide a subsection as above and focus on the study population, inclusion and exclusion criteria.

Sample size determination
The author(s) explained under a subsection titled 'Sample size determination' that 'The Cochran formulae (Cochran, 1977) … determined the sample size for the study…' (page 5). The author(s) should also seek to show the allocation of the proportional breakdown of the sample size to the ten hospitals in this subsection.

The author(s) noted that '…However, six hundred and seven (607) healthcare providers and ancillary staff participated in the study, resulting in a response rate of 93.1%. The main factor behind of not achieving 100% response rate was due to the lack of monetary compensation…' (page 5). Perhaps, this could be deferred till the first subsection titled 'socio-demographic characteristics of respondents' under the results section below.

Sampling method
The author(s) indicated under a subsection titled 'Sampling procedure' that 'A multistage sampling method comprising of selecting of districts, hospitals and study participants was employed.… Sampling methods applied in the study were simple random, stratified, and probability proportional to size…' (page 5). The author(s) should ensure a sequential presentation of the sampling methods applied related to these methods.

<u>Simple random</u>
The author(s) should seek to provide the theoretical definition and justification for the choice of 'Simple random' sampling used in this quantitative study. The practical application should be explained as well.

<u>Stratified sampling</u>
The author(s) should seek to provide the theoretical definition and justification for the choice of 'Stratified sampling' used in this quantitative study. The practical application should be explained as well.

<u>Probability proportional to size (sampling?)</u>
The author(s) should seek to provide the theoretical definition and justification for the choice of 'Probability proportional to size' sampling used in this quantitative study. The practical application should be explained as well.

Data collection
The author(s) presented a subsection titled 'Data collection' and stated that 'Structured questionnaire was used as the data collection tool for this study…' (pages 6-7).

The author(s) intimated that 'The questionnaire was structured into seven (5) [check this] sections namely: Section I: respondent's socio-demographic and lifestyle Characteristics, Section II: occupational factors; Section III: Organizational, behavioural and intervention

factors; Section IV: low back pain and Section V: turnover intention with 12, 11, 5, 2 and 2 questions, respectively…' (page 7). However, it is not clear why the 'lifestyle characteristics' were joined with the socio-demographic characteristics?

The author(s) should clarify which of these variables were considered as independent and dependent to respond to the earlier comment made under the objective of the study section above. Thus, there is a need for some clarification on which of the two suggested objectives that these variables may relate to. Perhaps, it would be suitable for the author(s) to state some of the questions asked with the responses stated as either 'Yes and No or Likert Scale'.

The author(s) should present the duration for administering each questionnaire. The author(s) should explain further how the interviewer-administered and self-administered strategies were applied in the administration of the questionnaires as well as the location of the interviews.

Quality assurance
The author(s) should seek to provide a subsection as above and a description of how data quality was assured in the study focusing on training of research assistants, pretesting of the questionnaire, validity and reliability of the instruments.

Data management and analysis
The author(s) provided a subsection titled 'Data management and analysis' and informed the reader how the data obtained from the returned questionnaires were analysed using the STATA SE version 15 (64-bit) statistical analysis software (34) for cleaning and analysis…' (pages 7-8).

The author(s) further explained that 'The descriptive statistics of the independent variables (socio-demographic and lifestyle characteristics, occupational factors, organizational, behavioural, and intervention strategies) were presented in the form of tables whereas that of the dependent variables (past 12-month exposure to low back pain) and duration of experiencing low back pain were presented in bar graph and pie chart. Statistical parameters including crude odds ratio, adjusted odds ratio, 95% confidence intervals and p-values were calculated based on a two sided test… A chi-square, fisher's exact and Mann-Whitney U tests were used to determine preliminary associations between prevalence of low back pain (prevalence of low back pain in past one year) and independent variables… Also, bivariate and multiple logistic regression was used to confirm the relationship between independent variables and prevalence of low back pain…' (pages 7-8).

While it is not clear why the 'lifestyle characteristics' were joined with the socio-demographic characteristics, the author(s) did not explain how the variable 'turnover intention' was measured in this study either as an independent variable or a dependent/outcome variable.

It was also not clear how and why the 'dependent variables' were described as 'past 12-month exposure to low back pain) and duration of experiencing low back pain'. Thus, it is not clear why there were 'associations between prevalence of low back pain (prevalence of low back pain in past one year) and independent variables but no association between low back pain and turnover intention; and independent variables and turnover intention (as dependent variable)'.

Thus, the author(s) should explain why turnover intention was not a dependent/outcome variable. The expectation would be that a health worker suffering from low back pain would wish to leave their job or position. Thus, there should be a relationship between low back pain and how the health workers leave their post. The key conclusion will depend on an association between low back pain and turnover intention. The current challenge will appear to affect the results obtained from the current data analysis.

If the author(s) will still use the 'predisposing (socio-demographic characteristics) factors' in the analysis, then the analysis should be based on the theoretical framework proposed by Andersen (1995). The second title and second objective suggested earlier could be considered to address this deficiency in the analysis. Generally, the author(s) should make strenuous efforts to clarify the independent and dependent variables of the study taking into account the objective(s) suggested earlier.

Ethical considerations

The author(s) explained under a subsection titled 'Ethical consideration' that 'The study protocol was approved by Committee on Human Research Publication and Ethics (CHRPE) of Kwame Nkrumah University of Science and Technology, Kumasi with an approval reference number, CHRPE/AP/807/22, and the Ghana Health Service Ethics Review Committee identity number GHS-ERC:012/03/23…Study respondents were briefed on potential risk/benefit, privacy and confidentiality, data storage and usage, voluntary consent/withdrawal, compensation, declaration of conflict of interest and research funding. Finally, participants read and completed a written informed consent form before permitted to take part in the study' (page 8).

Results

The author(s) presented the results of the study (pages 8-20). Despite the accuracy of the statistical results presented, an effort should be made to ensure a systematic presentation to bring it to the understanding of lay readers of the discipline.

Socio-demographic characteristics of respondents

The author(s) presented a subsection titled 'Socio-demographic and lifestyle characteristics of Health Workers' (page 8) supported by 'Table 1: Socio-demographic and lifestyle characteristics of health workers' (pages 9-10). The challenge still recurs regarding why the author(s) decided to incorporate 'lifestyle characteristics' into the socio-demographic characteristics of the respondents when lifestyle characteristics' should be a separate independent variable with distinct elements of measurement. The term that seems to be normally used is 'Behavioural and Lifestyle' factors when discussing risk factors for non-communicable diseases like hypertension and diabetes.

Occupation: It is not clear why 'Occupation' was used as an element of socio-demographic characteristics to describe the different professional groups within the health sector when the reader has already been informed that the study was conducted among health workers. Perhaps, 'professional category' will be appropriate within this context – hospital setting.

Type of health worker: It was not clear why the 'Type of health worker' was categorised into 'Healthcare provider and Ancillary staff'. Given the description within the Labour Laws and the formation of Labour Unions, there is a group known as Health Services Workers Union (mostly constituting administrative and ancillary staff of the health sector). Thus, it may not

be appropriate to use the description ‘healthcare provider’ without incorporating the ancillary staff. Every health worker contributes to healthcare provision/delivery because of the multi-purpose/task nature of the setting. Perhaps, a clear distinction will be to use ‘clinical personnel’ for professional health workers in this context.

The style of presentation where highest responses for different variables are picked without comparing the highest and lowest responses within the same variables makes it a bit difficult for the reader to follow. For instance, this statement will make the categorisation more confusing for the reader: ‘…the majority, 543 (89.3%) and 332 (54.7%) 258 belonged to healthcare provider category and nursing profession, respectively…’(page 8). These appear to relate to the ‘Type of health worker and Occupation’ as shown in Table 1 (pages 9-10).

The reader would have wished to see columns in the Table 1 been designed to take account of at least, the private hospitals and public hospitals where a breakdown will be shown for each of the variables measured respectively before the combined/total responses are provided, even if this cannot be done for each of the ten hospitals surveyed in the study.

The author(s) should be consistent in stating the decimal places in the proportions instead of using a mixture of one decimal and two decimal places concurrently. For instance, consider this section of the narrative ‘Most of the study respondents, 558 (91.93) had attained tertiary education whereas a greater portion, 283 (46.6%) had less than 5 years of working experience, and the median working experience was 5 years, with an interquartile range of 3 – 12 years. Also, 532 (87.6%) worked with public health facilities, and 512 (84.35) were permanent staff. Additionally, a greater number of respondents, 493 (81.22) worked for 5 days and below within a week. Besides, more than one-tenth, 100 (16.47) were supervisors’.

Prevalence of exposure to Low back pain among Health Workers
The author(s) presented a subsection titled ‘Prevalence of exposure to Low back pain among Health Workers’ (page 11) supported by ‘Fig 1: Prevalence of low back pain among health workers, and Fig 2: Duration of low back pain experienced by health workers’ (appendices). However, the title of figure 2 in the appendix is shown as ‘Fig 1: Duration of low back pain experienced by health workers’. This discrepancy should be corrected. Another difficulty relates to the use of ‘Prevalence of exposure…’ in the heading of the section ‘Prevalence of exposure to Low back pain among Health Workers’. It appears that the responses related to ‘Yes and No’ as presented in Figure 1. This means that the health workers had either already suffered or were not suffering from low back pain. However, the reader is not informed of how the ‘exposure’ was measured in the study. Perhaps, the author(s) could consider a title such as ‘Proportion of low back pain among health workers’ in this context since they had already suffered the back pain in this facility based study – it is not a community based study. Perhaps, this section could follow immediately after the section on ‘socio-demographic characteristics of respondents’.

Socio-demographic and lifestyle characteristics influencing Low back pain
The author(s) presented a subsection titled ‘Socio-demographic and lifestyle characteristics influencing Low back pain’ (page 11) supported by ‘Table 2: Socio-demographic and lifestyle characteristics influencing low back pain’ (pages 11-12). The author(s) narrated that ‘A significant association was found between age (t = - 3.57, p-value = < 0.01), working experience (t = - 2.41, p-value = 0.015), type of type of employment (Pearson chi square = 12.90, p-value = < 0.01), number of working days in a typical week (Pearson chi-square =

20.83, p-value = < 0.01), and exposure to low back pain (Table 3-17). Regarding lifestyle characteristics, frequency of exercise (Pearson chi-square = 4.53, p-value = 0.033) and daily hours of sleep (Pearson chi-square = 12.91, p-value = < 0.01) were found to be significantly related to low back pain exposure' (page 11). Perhaps, it will be easy for the reader to understand if the 'p-values' are stated as 'P<0.01 etc or P=0.001'.

A revision of the socio-demographic characteristics and lifestyle characteristics would affect the analysis in this section as well. Perhaps, this section could follow immediately after the section on 'prevalence/proportion of low back pain'.

Occupational related factors
The author(s) presented a subsection titled 'Occupational related factors' (page 10) without supporting the narrative with either a Table or Figure. This seems to be the descriptive statistics/results shown in 'Table 3: Occupational factors influencing low back pain exposure' (page 13). It will seem appropriate to join this narrative here with the narrative under the section titled 'Occupational factors influencing Low back pain Exposure' supported by Table 3 (pages 12-13). Technically, the variables measured as occupational related may fit within the organisational factors. This is because the author(s) did not specify what each of the individual respondents do within their profession as medical doctors, nurses, pharmacists, laboratory technologist etc. A revision of the occupational related factors would affect the analysis in this section as well.

Organizational, behavioural and intervention related factor
The author(s) presented a subsection titled 'Organizational, behavioural and intervention related factor' (pages 10-11) without supporting the narrative with either a Table or Figure. This seems to be the descriptive statistics/results shown in 'Table 4: Organizational, behavioural and intervention factors influencing low back pain exposure' (pages 13-14). It will seem appropriate to join this narrative here with the narrative under the section titled 'Organizational, behavioural and intervention factors influencing low back pain exposure' supported by Table 4 (page 13).

The confusion in this analysis also relates to the variables measured within the organisational, behavioural and intervention related factors. The variables measured as shown in the Table 4 were 'Understaffed, Use of work procedures, Trained on work machinery, Trained on transport aids, Trained on good working posture'. It was not immediately clear to the reader which of these belonged to 'organisational, behavioural and intervention' factors. Ideally, these three key factors should be seen as separate themes with their distinct elements of measurement. These should be seen as separate specific objectives that were used as independent variables in the study. Appropriate literature should be presented to demarcate how these factors were measured. A revision of the organizational, behavioural and intervention related factor would affect the analysis in this section as well.

Bivariate and multiple logistic regression of risk factors and exposure to low back pain
The author(s) presented a subsection titled 'Bivariate and multiple logistic regression of risk factors and exposure to low back pain' (page 16) supported by 'Table 5: Bivariate and multiple logistic regression of risk factors and exposure to low back pain' (pages 17-19). The author(s) explained that 'The table 5 below summarizes the bivariate and multiple logistic regression analysis between predisposing factors and exposure to low back pain...' (pages 15-16). There appears to be a confusion in the use of words such as 'risk factors' in the title,

‘exposure’ in the title and ‘predisposing’ in the narrative. The author(s) should reconcile the three words because using predisposing factors will connote socio-demographic characteristics when using the Andersen (1995) healthcare utilisation model. A revision of what constitutes, socio-demographic characteristics, organizational factors, behavioural and lifestyle factors, and intervention related factors would affect the analysis in this section as well.

Turnover Intention Effects of Low back pain on Health Workers
The author(s) presented a subsection titled ‘Turnover Intention Effects of Low back pain on Health Workers’ (page 20) supported by ‘Table 6: Turnover Intention Effects of Low back pain on Health Workers’ (page 20). The author(s) explained that ‘The table 6 depicts the turnover intention effects of study participants after their experience of low back pain. Out of the 495 (81.6%) that experienced low back pain, a substantial number, 123 (24.9%) and 144 (29.1%) occasionally considers leaving their job, and willing to accept another job at same compensation, respectively’ (page 20).

This is where the misunderstanding of the dependent/outcome variable of the study pops up. The reader has not been told anywhere in the report how turnover intention was measured/what constituted turnover intention. The statements made in Table 6 did not ask the respondents whether they would stay or leave because of low back pain. This would have shown the proportion of respondents who had intended to leave or stay. A reader would have wished to see a follow up question which solicited the reasons that might account for this decision (this could have included the low back pain). Even if the current analysis was considered appropriate, just using proportions could not be seen as a strong basis for the relationship that is supposed to be established between low back pain and turnover intention among the health workers.

The reader would expect to see an association between low back pain (intermediate variable) and turnover intention (dependent variable). The key conclusion will depend on an association between low back pain and turnover intention and the related factors. This would have been preceded by an analysis of the relationships between socio-demographic characteristics, organisational factors, behavioural and lifestyle factors, intervention strategies and turnover intention. An analysis of this nature will be more convincing. Thus, a revision of what constitutes, socio-demographic characteristics, organizational factors, behavioural and lifestyle factors, and intervention related factors would affect the analysis in this section as well.

Discussion
The author(s) presented a section titled ‘Discussion’ and explained among other things, that ‘In this present study, a past 12-month prevalence of 81.6% was reported. This was parallel to studies conducted in Slovenia (85.9%) (35), Saudi Arabia (81.4%) (36) and China (80.0%) (37)…’ (pages 20-23). The author(s) should seek to provide the analysis under subsections related to the key variables of interest (when the entire study is revised). There should be consistency in the presentation to ensure an easy flow of ideas for the reader. This will be achieved when the findings have been related to the key elements of the objective(s), existing literature and possibly, the theoretical perspective (when applied).

Conclusion
The author(s) presented a section titled 'Conclusion' and indicated among other things, that 'The magnitude of low back pain among healthcare providers and ancillary staff in the Greater Accra region was high.…' (pages 23-24). The conclusion may be affected by a revision of the entire work. The author(s) should seek to re-arrange the section to include subsections on implications for policy and practice, and limitations to the study/future research as follows.

Implications for Policy and Practice
The author(s) should seek to present a subsection titled 'Implications for Policy and Practice'. Here, suggestions could be made for policy makers and practitioners in the health sector based on the findings of the study.

Limitations to the study and future research
The author(s) presented the limitations to the study under a section titled 'Strength and limitations' (page 23). The author(s) should seek to present suggestions for future research in addition to the limitations stated.

Other Issues
The author(s) could ensure proof reading of the manuscript.
