# Supplementary material for: Prevalence, predisposing factors, and turnover intention related to low back pain among health workers in Accra, Ghana
Source: PLoS One. 2025 Jan 16;20(1):e0317582. doi: 10.1371/journal.pone.0317582 (PMC11737759; doi:10.1371/journal.pone.0317582)
Supplement: Attachment — Submitted filename: Response to reviewers.docx [file pone.0317582.s004.docx]

**Manuscript ID:** PONE-D-23-26548

**Manuscript Title:** Prevalence, predisposing factors, and turnover intention related to low back pain among health workers in Accra, Ghana

| **Reviewer 1 comments** | **Authors’ response** | **Page**  **number** |
| --- | --- | --- |
| Objective(s) of the study  The author(s) indicated in the abstract that ‘This study investigated the prevalence,  predisposing factors, and turnover intention effects of LBP among health workers in Ghana.’  (page 2).  The author(s) stated in the introduction section that ‘This study, therefore, investigated the  prevalence, predisposing factors and turnover intention effects of low back pain among  healthcare providers and ancillary staff in the Greater Accra region of Ghana’ (page 4).  The author(s) should ensure consistency in the statement of the objectives in the abstract, and the introduction section. More importantly, deducing from the two objectives stated above, there is a lack of clarity on the focus of the study – what is the dependent or outcome variable of the study? There is also a lack of coherence between the title of the paper and the objective of the study. The title of the paper is ‘Predisposing factors and turnover intention effects of low back pain among health workers in selected hospitals in the Greater Accra region, Ghana’ This account for the difficult in devising an objective to reflect what the study sought to achieve. Perhaps, the above challenge(s) could be addressed if the title is rephrased. For instance, the author(s) could consider the following; 1. ‘Effects of low back pain on turnover intention among health workers in selected hospitals in the Greater Accra Region, Ghana’ 2. ‘Factors associated with low back pain and its effect on turnover intention among health workers in selected hospitals in the Greater Accra Region, Ghana’ The objectives related to the above suggested titles could be stated as: 1. ‘To determine the effects of low back pain on turnover intention among health workers in selected hospitals in the Greater Accra Region, Ghana’ 2. ‘To assess factors associated with low back pain and its effect on turnover intention among health workers in selected hospitals in the Greater Accra Region, Ghana’ For the purpose of the comments in this report, the second title and second objective stated above would be considered. This will mean that the dependent variable or outcome will be turnover intention. In this case, low back pain could be considered as the intermediate variable to turnover intention. | The objective has been clarified to make our dependent variable (low back pain) come out clearer. Also, its consistency has been ensured in both the abstract and introduction.  The title of the paper has also been rephrased to capture the content of the paper, making the dependent variable still remaining low back pain. | 2-4 |
| **Restructuring the manuscript** Unless it is the requirement of the journal, the author(s) could restructure the manuscript as below to ensure that the reader is able to follow the line of argument with ease. **Introduction** The author(s) presented a section titled above where there was an analysis of extant literature on the topic under discussion (pages 3-4). Perhaps, a separation of the introduction from the literature review and theoretical perspective would make it easy for the reader as well. Thus, the author(s) should seek to present an analysis of the problem, limitations in the extant literature and provide the justification for the current study (as partly done) in the introduction section here. The research objective should follow from the above as stated in the concluding paragraph of the section (this should take account of the needed revision to be made).  **Literature review** There should be a section as above and be re-arranged to let the reader know what the literature review part is, and what the theoretical perspective part is, as suggested below.  *Literature review of related studies* There should be a part of the section that explains to the reader what has been written about the topic and the concepts therein. For instance, there should be a clearer analysis of studies related to prevalence/proportion of low back pain among health workers, and prevention/proportion of turnover intention among health workers. There should also be an analysis of studies related to how predisposing (socio-demographic characteristics) factors, organisational factors, lifestyle factors, behavioural factors and intervention factors have been associated with low back pain and turnover intention among health workers. These concepts should come out clearly (even if presented in paragraphs) in the write up. *Theoretical perspective* Even though the author(s) indicated that there were predisposing factors associated with low back pain and turnover intention (emphasis mine), there was no theoretical foundation stated. Perhaps, the Andersen (1995) healthcare utilisation model which projects predisposing factors, enabling factors and need factors could be considered. This could help to show a linkage between the socio-demographic characteristics as the predisposing factors, the organisational factors as enabling factors and lifestyle/behavioural factors as the need factors in a conceptual framework. The author(s) should ensure that the tenets of the chosen theory/theories are properly explained and supported by studies that have applied them before (even if presented in paragraphs) in the write up. **Methods** The author(s) presented a section titled ‘Materials and Methods’ with subsections (pages 4- 8). The author(s) should seek to revise the section to portray the following key subsections. *Study Design* The author(s) presented a subsection titled ‘Study design, participants and setting’ and stated that ‘An analytic facility-based cross sectional study design with a quantitative approach was employed for this research’ (page 4). This section should focus on the study design as titled above. The author(s) should present a theoretical definition a justification for the choice of this study design as well as the quantitative research method applied. *Study Area* The author(s) presented the study area as part of a subsection titled ‘Study design, participants and setting’ and stated that ‘The study was carried … ten (10) hospitals in the Greater Accra region of Ghana… The Greater Accra region is one of the sixteen regions of Ghana (22) … The region is regarded as an area with the highest population density due to its high in-migration and population growth rate (24)’ (pages 4-5). This description failed to provide the international reader with a clearer idea of the socioeconomic challenges impeding access to healthcare among people in the study area. The author(s) should seek to explain to the reader further background of the Greater Accra Region and Ghana in which these hospitals are located in terms of geography, demography, economic status - before the description of health care provision (in sequence/paragraphs). This analysis will enable the international reader to understand the setting of the study and the justification for the choice based on these backgrounds. These should precede the description of the selected hospitals.  *Study population* The author(s) presented the study population as part of a subsection titled ‘Study design, participants and setting’ and stated that ‘The study was carried out among physicians, nurses, midwives, medical laboratory staff, physiotherapists, health care assistants, orderlies and laundry workers of ten (10) hospitals in the Greater Accra region of Ghana’ (page 4). The author(s) should provide a subsection as above and focus on the study population, inclusion and exclusion criteria. *Sample size determination* The author(s) explained under a subsection titled ‘Sample size determination’ that ‘The Cochran formulae (Cochran, 1977) … determined the sample size for the study…’ (page 5). The author(s) should also seek to show the allocation of the proportional breakdown of the sample size to the ten hospitals in this subsection. The author(s) noted that ‘…However, six hundred and seven (607) healthcare providers and ancillary staff participated in the study, resulting in a response rate of 93.1%. The main factor behind of not achieving 100% response rate was due to the lack of monetary compensation…’ (page 5). Perhaps, this could be deferred till the first subsection titled ‘socio-demographic characteristics of respondents’ under the results section below. *Sampling method* The author(s) indicated under a subsection titled ‘Sampling procedure’ that ‘A multistage sampling method comprising of selecting of districts, hospitals and study participants was employed.… Sampling methods applied in the study were simple random, stratified, and probability proportional to size…’ (page 5). The author(s) should ensure a sequential presentation of the sampling methods applied related to these methods. Simple random The author(s) should seek to provide the theoretical definition and justification for the choice of ‘Simple random’ sampling used in this quantitative study. The practical application should be explained as well. Stratified sampling The author(s) should seek to provide the theoretical definition and justification for the choice of ‘Stratified sampling’ used in this quantitative study. The practical application should be explained as well. Probability proportional to size (sampling?) The author(s) should seek to provide the theoretical definition and justification for the choice of ‘Probability proportional to size’ sampling used in this quantitative study. The practical application should be explained as well. *Data collection* The author(s) presented a subsection titled ‘Data collection’ and stated that ‘Structured questionnaire was used as the data collection tool for this study…’ (pages 6-7). The author(s) intimated that ‘The questionnaire was structured into seven (5) [check this] sections namely: Section I: respondent’s socio-demographic and lifestyle Characteristics, Section II: occupational factors; Section III: Organizational, behavioural and intervention  factors; Section IV: low back pain and Section V: turnover intention with 12, 11, 5, 2 and 2 questions, respectively…’ (page 7). However, it is not clear why the ‘lifestyle characteristics’ were joined with the socio-demographic characteristics? The author(s) should clarify which of these variables were considered as independent and dependent to respond to the earlier comment made under the objective of the study section above. Thus, there is a need for some clarification on which of the two suggested objectives that these variables may relate to. Perhaps, it would be suitable for the author(s) to state some of the questions asked with the responses stated as either ‘Yes and No or Likert Scale’. The author(s) should present the duration for administering each questionnaire. The author(s) should explain further how the interviewer-administered and self-administered strategies were applied in the administration of the questionnaires as well as the location of the interviews. *Quality assurance* The author(s) should seek to provide a subsection as above and a description of how data quality was assured in the study focusing on training of research assistants, pretesting of the questionnaire, validity and reliability of the instruments. *Data management and analysis* The author(s) provided a subsection titled ‘Data management and analysis’ and informed the reader how the data obtained from the returned questionnaires were analysed using the STATA SE version 15 (64-bit) statistical analysis software (34) for cleaning and analysis…’ (pages 7-8). The author(s) further explained that ‘The descriptive statistics of the independent variables (socio-demographic and lifestyle characteristics, occupational factors, organizational, behavioural, and intervention strategies) were presented in the form of tables whereas that of the dependent variables (past 12-month exposure to low back pain) and duration of experiencing low back pain were presented in bar graph and pie chart. Statistical parameters including crude odds ratio, adjusted odds ratio, 95% confidence intervals and p-values were calculated based on a two sided test… A chi-square, fisher’s exact and Mann-Whitney U tests were used to determine preliminary associations between prevalence of low back pain (prevalence of low back pain in past one year) and independent variables… Also, bivariate and multiple logistic regression was used to confirm the relationship between independent variables and prevalence of low back pain…’ (pages 7-8). While it is not clear why the ‘lifestyle characteristics’ were joined with the sociodemographic characteristics, the author(s) did not explain how the variable ‘turnover intention’ was measured in this study either as an independent variable or a dependent/outcome variable. It was also not clear how and why the ‘dependent variables’ were described as ‘past 12- month exposure to low back pain) and duration of experiencing low back pain’. Thus, it is not clear why there were ‘associations between prevalence of low back pain (prevalence of low back pain in past one year) and independent variables but no association between low back pain and turnover intention; and independent variables and turnover intention (as dependent variable)’.  Thus, the author(s) should explain why turnover intention was not a dependent/outcome variable. The expectation would be that a health worker suffering from low back pain would wish to leave their job or position. Thus, there should be a relationship between low back pain and how the health workers leave their post. The key conclusion will depend on an association between low back pain and turnover intention. The current challenge will appear to affect the results obtained from the current data analysis. If the author(s) will still use the ‘predisposing (socio-demographic characteristics) factors’ in the analysis, then the analysis should be based on the theoretical framework proposed by Andersen (1995). The second title and second objective suggested earlier could be considered to address this deficiency in the analysis. Generally, the author(s) should make strenuous efforts to clarify the independent and dependent variables of the study taking into account the objective(s) suggested earlier.  *Ethical considerations* The author(s) explained under a subsection titled ‘Ethical consideration’ that ‘The study protocol was approved by Committee on Human Research Publication and Ethics (CHRPE) of Kwame Nkrumah University of Science and Technology, Kumasi with an approval reference number, CHRPE/AP/807/22, and the Ghana Health Service Ethics Review Committee identity number GHS-ERC:012/03/23…Study respondents were briefed on potential risk/benefit, privacy and confidentiality, data storage and usage, voluntary consent/withdrawal, compensation, declaration of conflict of interest and research funding. Finally, participants read and completed a written informed consent form before permitted to take part in the study’ (page 8). | Usually, manuscript presents a concise yet summarized research. Authors respectfully cannot restructure the manuscript into the proposed structure. The proposed structure looks more like a main thesis format than a manuscript, and may require readers to read bits and pieces of information before getting to the climax of the work. However, some of the concerns raised have been addressed.  The literature review and theoretical review have been fused in the introduction. The section has been improved to reflect the theoretical review aspect of the work. The authors don’t see the need to separate them because in the reading of a manuscript, the audience are expected to read a short and precise introduction section that sets the tone for reading of the work. Separating these sections is not encouraged in manuscripts.  Respectfully, this is a manuscript and does not necessarily require writing the definitions of study design used in it. The authors don’t see how the socioeconomic challenges impending access to healthcare among people link to a study on low back pain among health workers.  The inclusion and exclusion criteria were presented after the sampling procedure, which the authors see it to be appropriate.  This is the actual thesis and not a proposal. Again, authors don’t see why a breakdown of sample size at the proposal stage is necessary here in a manuscript from a final thesis work.  The authors necessarily don’t need to state reasons for all the sampling procedures used in the manuscript. There are reasons why these sampling methods are used in a plethora of literature. In cases, reviewers want to know, authors may provide the reasons but not necessarily written in the manuscript.  Few questions were captured under lifestyle characteristics. These were merged just to let readers know some of the questions are lifestyle focused, and to prevent pieces of information in the same manuscript. The headings have been written in the table to show the difference between these two characteristics, socio-demographic and lifestyle.  The sections that were used as independent variables have clarified in the manuscript, and same applies to the dependent variables. The study questionnaire was attached to the manuscript. So, it is not important to state some of the questions and their scale of measurement or responses in the manuscript. However, the outcome variable, low back pain, and their responses have been defined in the manuscript.  The time and strategy for administration of questionnaire has already been stated in the manuscript. The location of interviews has been added as suggested.  Turnover intention and duration of low back pain were just follow-up questions to the outcome variable, low back pain. This has been specified in the data analysis method. So, the current data just suggested that some study participants had intention of leaving their work after experiencing low back pain, and not an association between the two variables. Therefore, the dependent variable remains low back pain. | 2-25 |
| The author(s) presented the results of the study (pages 8-20). Despite the accuracy of the statistical results presented, an effort should be made to ensure a systematic presentation to bring it to the understanding of lay readers of the discipline. *Socio-demographic characteristics of respondents* The author(s) presented a subsection titled ‘Socio-demographic and lifestyle characteristics of Health Workers’ (page 8) supported by ‘Table 1: Socio-demographic and lifestyle characteristics of health workers’ (pages 9-10). The challenge still recurs regarding why the author(s) decided to incorporate ‘lifestyle characteristics’ into the socio-demographic characteristics of the respondents when lifestyle characteristics’ should be a separate independent variable with distinct elements of measurement. The term that seems to be normally used is ‘Behavioural and Lifestyle’ factors when discussing risk factors for noncommunicable diseases like hypertension and diabetes. Occupation: It is not clear why ‘Occupation’ was used as an element of socio-demographic characteristics to describe the different professional groups within the health sector when the reader has already been informed that the study was conducted among health workers. Perhaps, ‘professional category’ will be appropriate within this context – hospital setting. Type of health worker: It was not clear why the ‘Type of health worker’ was categorised into ‘Healthcare provider and Ancillary staff’. Given the description within the Labour Laws and the formation of Labour Unions, there is a group known as Health Services Workers Union (mostly constituting administrative and ancillary staff of the health sector). Thus, it may not 7 be appropriate to use the description ‘healthcare provider’ without incorporating the ancillary staff. Every health worker contributes to healthcare provision/delivery because of the multipurpose/task nature of the setting. Perhaps, a clear distinction will be to use ‘clinical personnel’ for professional health workers in this context. The style of presentation where highest responses for different variables are picked without comparing the highest and lowest responses within the same variables makes it a bit difficult for the reader to follow. For instance, this statement will make the categorisation more confusing for the reader: ‘…the majority, 543 (89.3%) and 332 (54.7%) 258 belonged to healthcare provider category and nursing profession, respectively…’(page 8). These appear to relate to the ‘Type of health worker and Occupation’ as shown in Table 1 (pages 9-10). The reader would have wished to see columns in the Table 1 been designed to take account of at least, the private hospitals and public hospitals where a breakdown will be shown for each of the variables measured respectively before the combined/total responses are provided, even if this cannot be done for each of the ten hospitals surveyed in the study. The author(s) should be consistent in stating the decimal places in the proportions instead of using a mixture of one decimal and two decimal places concurrently. For instance, consider this section of the narrative ‘Most of the study respondents, 558 (91.93) had attained tertiary education whereas a greater portion, 283 (46.6%) had less than 5 years of working experience, and the median working experience was 5 years, with an interquartile range of 3 – 12 years. Also, 532 (87.6%) worked with public health facilities, and 512 (84.35) were permanent staff. Additionally, a greater number of respondents, 493 (81.22) worked for 5 days and below within a week. Besides, more than one-tenth, 100 (16.47) were supervisors’. | As already indicated, few questions were used to assess lifestyle characteristics pertaining to low back pain, that’s why they were merged to prevent too much bits and pieces in the work. However, the separation has been indicated in the same table.  Professional category has been used to fit the context.  Clinical staff and supporting staff have been used to replace healthcare providers (though healthcare providers are also used) and ancillary staff, respectively.  The narratives are written to highlight the information in the tables. Also, the highest responses tell a better story than narrating the lowest as part. The table is there as reference to the narration, and not to narrate most of its content.  A breakdown for private and public is not necessary because the study is not a comparative one between workers in private and public hospitals.  The percentages have been rounded to 1d.p. | 8-20 |
| *Prevalence of exposure to Low back pain among Health Workers* The author(s) presented a subsection titled ‘Prevalence of exposure to Low back pain among Health Workers’ (page 11) supported by ‘Fig 1: Prevalence of low back pain among health workers, and Fig 2: Duration of low back pain experienced by health workers’ (appendices). However, the title of figure 2 in the appendix is shown as ‘Fig 1: Duration of low back pain experienced by health workers’. This discrepancy should be corrected. Another difficulty relates to the use of ‘Prevalence of exposure…’ in the heading of the section ‘Prevalence of exposure to Low back pain among Health Workers’. It appears that the responses related to ‘Yes and No’ as presented in Figure 1. This means that the health workers had either already suffered or were not suffering from low back pain. However, the reader is not informed of how the ‘exposure’ was measured in the study. Perhaps, the author(s) could consider a title such as ‘Proportion of low back pain among health workers’ in this context since they had already suffered the back pain in this facility based study – it is not a community based study. Perhaps, this section could follow immediately after the section on ‘socio-demographic characteristics of respondents’. | The title of the section and caption of the figure have been corrected to ‘proportion of low back pain’ to prevent any discrepancies. | 12 |
| *Occupational related factors* The author(s) presented a subsection titled ‘Occupational related factors’ (page 10) without supporting the narrative with either a Table or Figure. This seems to be the descriptive statistics/results shown in ‘Table 3: Occupational factors influencing low back pain exposure’ (page 13). It will seem appropriate to join this narrative here with the narrative under the section titled ‘Occupational factors influencing Low back pain Exposure’ supported by Table 3 (pages 12-13). Technically, the variables measured as occupational related may fit within the organisational factors. This is because the author(s) did not specify what each of the individual respondents do within their profession as medical doctors, nurses, pharmacists, laboratory technologist etc. A revision of the occupational related factors would affect the analysis in this section as well. | The narration of the subsection title, ‘occupational related factors’ has now been supported with a table. | 10 |
| *Organizational, behavioural and intervention related factor* The author(s) presented a subsection titled ‘Organizational, behavioural and intervention related factor’ (pages 10-11) without supporting the narrative with either a Table or Figure. This seems to be the descriptive statistics/results shown in ‘Table 4: Organizational, behavioural and intervention factors influencing low back pain exposure’ (pages 13-14). It will seem appropriate to join this narrative here with the narrative under the section titled ‘Organizational, behavioural and intervention factors influencing low back pain exposure’ supported by Table 4 (page 13). The confusion in this analysis also relates to the variables measured within the organisational, behavioural and intervention related factors. The variables measured as shown in the Table 4 were ‘Understaffed, Use of work procedures, Trained on work machinery, Trained on transport aids, Trained on good working posture’. It was not immediately clear to the reader which of these belonged to ‘organisational, behavioural and intervention’ factors. Ideally, these three key factors should be seen as separate themes with their distinct elements of measurement. These should be seen as separate specific objectives that were used as independent variables in the study. Appropriate literature should be presented to demarcate how these factors were measured. A revision of the organizational, behavioural and intervention related factor would affect the analysis in this section as well | The narrative has been supported with a table. The variables have been differentiated in the table to make it clearer. Literature has been provided in the background that provides information supporting these factors in general and not all the specifics studied in the manuscript since this will be necessary in a thesis work and not manuscript. | 11 |
| *Bivariate and multiple logistic regression of risk factors and exposure to low back pain* The author(s) presented a subsection titled ‘Bivariate and multiple logistic regression of risk factors and exposure to low back pain’ (page 16) supported by ‘Table 5: Bivariate and multiple logistic regression of risk factors and exposure to low back pain’ (pages 17-19). The author(s) explained that ‘The table 5 below summarizes the bivariate and multiple logistic regression analysis between predisposing factors and exposure to low back pain...’ (pages 15- 16). There appears to be a confusion in the use of words such as ‘risk factors’ in the title, 9 ‘exposure’ in the title and ‘predisposing’ in the narrative. The author(s) should reconcile the three words because using predisposing factors will connote socio-demographic characteristics when using the Andersen (1995) healthcare utilisation model. A revision of what constitutes, socio-demographic characteristics, organizational factors, behavioural and lifestyle factors, and intervention related factors would affect the analysis in this section as well. | ‘Predisposing factors’ has been used throughout the manuscript to ensure consistency. | 15-19 |
| *Turnover Intention Effects of Low back pain on Health Workers* The author(s) presented a subsection titled ‘Turnover Intention Effects of Low back pain on Health Workers’ (page 20) supported by ‘Table 6: Turnover Intention Effects of Low back pain on Health Workers’ (page 20). The author(s) explained that ‘The table 6 depicts the turnover intention effects of study participants after their experience of low back pain. Out of the 495 (81.6%) that experienced low back pain, a substantial number, 123 (24.9%) and 144 (29.1%) occasionally considers leaving their job, and willing to accept another job at same compensation, respectively’ (page 20). This is where the misunderstanding of the dependent/outcome variable of the study pops up. The reader has not been told anywhere in the report how turnover intention was measured/what constituted turnover intention. The statements made in Table 6 did not ask the respondents whether they would stay or leave because of low back pain. This would have shown the proportion of respondents who had intended to leave or stay. A reader would have wished to see a follow up question which solicited the reasons that might account for this decision (this could have included the low back pain). Even if the current analysis was considered appropriate, just using proportions could not be seen as a strong basis for the relationship that is supposed to be established between low back pain and turnover intention among the health workers. The reader would expect to see an association between low back pain (intermediate variable) and turnover intention (dependent variable). The key conclusion will depend on an association between low back pain and turnover intention and the related factors. This would have been preceded by an analysis of the relationships between socio-demographic characteristics, organisational factors, behavioural and lifestyle factors, intervention strategies and turnover intention. An analysis of this nature will be more convincing. Thus, a revision of what constitutes, socio-demographic characteristics, organizational factors, behavioural and lifestyle factors, and intervention related factors would affect the analysis in this section as well. | The title of the subsection has been modified to reflect the exact position of the paper.  The outcome variable remains low back pain and not turnover intention as shown in the revised manuscript.  So, turnover intention was rather a follow up question after the outcome variable (low back pain), meaning workers having the intention of leaving their jobs after experiencing low back pain. This was not meant to establish an association between the two variables. The study recommended further on that aspect of the work. | 20 |
| **Discussion** The author(s) presented a section titled ‘Discussion’ and explained among other things, that ‘In this present study, a past 12-month prevalence of 81.6% was reported. This was parallel to studies conducted in Slovenia (85.9%) (35), Saudi Arabia (81.4%) (36) and China (80.0%) (37)…’ (pages 20-23). The author(s) should seek to provide the analysis under subsections related to the key variables of interest (when the entire study is revised). There should be consistency in the presentation to ensure an easy flow of ideas for the reader. This will be achieved when the findings have been related to the key elements of the objective(s), existing literature and possibly, the theoretical perspective (when applied) | The discussion was presented based on key findings of the research. An introductory paragraph has been created to present the key findings before the subsequent discussions. Each subsequent paragraph discusses one or two of the main findings. | 20-24 |
| **Conclusion** The author(s) presented a section titled ‘Conclusion’ and indicated among other things, that ‘The magnitude of low back pain among healthcare providers and ancillary staff in the Greater Accra region was high.…’ (pages 23-24). The conclusion may be affected by a revision of the entire work. The author(s) should seek to re-arrange the section to include subsections on implications for policy and practice, and limitations to the study/future research as follows. *Implications for Policy and Practice* The author(s) should seek to present a subsection titled ‘Implications for Policy and Practice’. Here, suggestions could be made for policy makers and practitioners in the health sector based on the findings of the study. *Limitations to the study and future research* The author(s) presented the limitations to the study under a section titled ‘Strength and limitations’ (page 23). The author(s) should seek to present suggestions for future research in addition to the limitations stated. | The conclusion will not change based on the outcome variable still being low back pain.  A paragraph on ‘Implication for policy and practice’ has been added to the discussion section of the study.  Also, a limitation to the study section was already in the manuscript but has been improved. | 24 |
| **Other Issues** The author(s) could ensure proof reading of the manuscript. | The manuscript has been proofread to correct all errors. | 2-25 |

| **Reviewer 2 comments** | **Authors’ response** | **Page**  **number** |
| --- | --- | --- |
| Please note that in a cross-sectional design, causality cannot be established. Therefore, it would be more accurate to describe the observed relationship between turnover intention and low back pain as an association rather than an effect. Please, follow the same fashion throughout the manuscript. | Questions based on turnover intention were used as a follow up to find out from victims of low back pain if they want to leave their jobs because of the pain. Statistically, it was not possible to establish association between low back pain and turnover intention since not all participants were asked these follow up questions. However, the word ‘effect’ has been removed from the manuscript, and subsequently, the topic has been reworded to reflect the content of the manuscript. | 1-23 |
| Please clarify the abbreviation 'CI' for the readers. It stands for Confidence Interval. For instance, the reported prevalence of LBP was 81.6% [95% CI: (78.2% - 84.6%)]. Including the full form at least once at the beginning would enhance the clarity of your findings for the readers. | Confidence interval (CI) has been defined at the first mention in the main manuscript, and the abbreviation had been used afterwards. Nonetheless, the full form of CI was not used in the abstract because CI are recognized statistical abbreviation used in reporting. Additionally, the abstract is just a glimpse or summary of the study with limited count of words, and may not accommodate full forms of all abbreviations like the CI, COR, AOR and others. | 1-23 |
| Please specify the unit for 'Sleep duration ≥ 8' to enhance clarity. For example, is it in hours? Providing this detail would help readers better understand the reported Adjusted Odds Ratio (AOR) of 0.54 (0.31, 0.97). | The variable was captured ‘daily hours of sleep’, which shows that unit of sleep is measured in hours. Footnotes are already captured under the table and it provides full meaning of AOR, COR and others. | 18 |
| Consider specifying the type of interventions to be implemented in these areas to address the burden of LBP. Additionally, elaborating on the proposed mechanisms through which these interventions would operate and identifying the target population for these interventions would enhance the clarity and applicability of your recommendations. | A paragraph on implication to practice and policy has been added to the discussion section to highlight study findings as well as the interventions and related mechanisms of implementation. | 23 |
| Describing LBP as 'one of the most severe life-threatening burdens' of noncommunicable conditions may be overstated. While LBP is  significant, it's important to maintain accuracy in descriptions. Noncommunicable diseases encompass a wide range of conditions, and LBP is not typically life-threatening like cardiovascular diseases or cancer. | The sentence has been paraphrased to reflect an accurate account of the severity of the condition. | 3 |
| In the introduction, it would be beneficial to adopt a consistent approach with abbreviations. After the first mention of each term, using the abbreviated form subsequently can streamline the text and improve readability. For example, after introducing 'low back pain (LBP)', subsequent references can use the abbreviation 'LBP'. | The correction has been affected and used throughout the manuscript. | 2-23 |
| Consider refraining from using colloquial expressions like 'quite unfortunately' in academic writing. Instead, opt for more formal and precise language to maintain professionalism and clarity in your text. | The word ‘quite unfortunately’ has been reworded to ‘unfortunately’ to maintain a formal and concise language. | 3 |
| Reporting and comparing different types of low back pain as simply 'low back pain' could be misleading. Define the specific types studied and focus on relevant literature to ensure accuracy and clarity. | Based on our findings, the different types of low back pain have been incorporated in the work (Fig. 2). | 10 |
| Clarify the necessity of studying predisposing factors and turnover intention in your setting. Providing this rationale strengthens the significance and relevance of your study. | The introduction has been modified to include this rationale. | 2 |
| Please specify the definition of healthcare professionals used in your study for clarity. | The term has been clarified in the methods section. A running term, health workers, has been used throughout the study. | 4 |
| Given that you have access to statistics regarding the number of healthcare professionals, it would be appropriate to apply the Cochrane formula with a finite population correction. This adjustment accounts for the specific population size being studied, ensuring more accurate sample size calculations. Please consider referencing the appropriate formula and methodology to support your sample size determination. | The sample size estimation considered Neyman’s finite population correction, which is similar to Cochrane correction formulae. Also, the sample size was raised to this current number due to the use of design effect. All references have been revised and cited appropriately. | 5 |
| Specify the key stakeholders involved in the review process for clarity. Additionally, clarify whether the participants of the pre-test were  included or excluded from the subsequent analysis to avoid ambiguity. | The stakeholders have been clarified. The pre-testing participants were excluded from analysis, and has been clarified. | 7 |
| Caution is advised when admonishing participants to complete questionnaires quickly, as rushed responses may lead to inaccuracies  or misinformation. Providing adequate time for thoughtful consideration can enhance the quality and reliability of the data collected. | The statement has been modified since most participants completed the questionnaire by the end of the next day. Participants were admonished to complete the questionnaire as early as possible because if two much time is given, participants end up misplacing the questionnaire. | 6 |
| Could you please provide information on the reliability and validity checking procedures conducted for the study? Reporting these measures would enhance the credibility and robustness of your findings. | No validity and reliability variables were measured. However, the questionnaire designed for already validated questionnaire, and it was subjected to pilot test as earlier mentioned to access face and content validity as part of the research process. | 6 |
| Consider streamlining the methodology section by avoiding repetitive information and focusing on essential details. Prioritize concise descriptions that convey the key aspects of the study design, procedures, and analyses, ensuring clarity without unnecessary repetition. | The methodology section has been paraphrased and modified to avoid repetition. | 4-7 |
| It appears that some percentages in Table 1 are missing the '%' sign. Please double-check the reported percentages against your output to ensure accuracy and consistency throughout the table. | The ‘%’ was written once in the heading of the table 1 to represent the reported percentages. | 8 |
| Could you please provide the rationale behind the classification of variables such as age, highest education level, working experience, type of health facility, type of employment, current position, daily hours of sleep, and working days in a typical week? | Classifying age allows researchers to analyze potential age-related patterns or differences in the prevalence of LBP and its associated factors.  Higher education levels may indicate better access to healthcare information or resources, potentially influencing LBP outcomes.  Longer working experience may suggest increased exposure to occupational hazards or ergonomic risk factors.  Classifying health facilities (public and private hospitals) helps assess whether differences in facility type influence LBP prevalence. Variations in resources, workload, and workplace policies between facility types may affect the prevalence of LBP among healthcare workers.  Different types of employment (e.g., permanent and temporary) may entail varying job demands, benefits, and occupational risks, which can influence LBP prevalence. Classifying employment types allows researchers to examine how employment status relates to LBP outcomes.  Categorizing current positions (Supervisor, Head of Department and No position) helps explore how job roles and responsibilities contribute to LBP prevalence. Certain positions may involve more physically demanding tasks or prolonged periods of sitting or standing, impacting LBP risk.  Daily hours of sleep: Classifying daily sleep duration (e.g., 8 hours and below, >8 hours) allows researchers to investigate the association between sleep patterns and LBP prevalence. Inadequate or excessive sleep duration may affect musculoskeletal health and contribute to LBP development.  Categorizing weekly working days (e.g., 5 days and below and > 5 days) helps assess the relationship between workload and LBP prevalence. Longer workweeks may result in increased physical strain and fatigue, potentially influencing LBP risk among healthcare workers. |  |
| Could you please clarify how you measured awkward posture or good posture in your study? Additionally, for the reported data on Occupational related factors, did you assess the experience over a certain period or the entire service life of the participants? | Participants were asked to self-report their typical posture during work hours. And subsequently asked how often they need to work in other postures. All occupational factors were assessed within the past one year. |  |
| In the discussion section, it's advisable to provide an overview of your findings at the beginning. This summary helps orient readers and provides context for the subsequent detailed analysis and interpretation of your results. | An overview of the findings has been added. | 19 |
| Consider providing a more logical explanation in your discussion section by incorporating region-specific studies to support or explain your findings. Focusing on research conducted in the same geographic area can enhance the relevance and applicability of your  study's conclusions | Studies from same geographical locations have been included in the discussion. | 19-23 |
