# Supplementary material for: Prevalence, predisposing factors, and turnover intention related to low back pain among health workers in Accra, Ghana
Source: PLoS One. 2025 Jan 16;20(1):e0317582. doi: 10.1371/journal.pone.0317582 (PMC11737759; doi:10.1371/journal.pone.0317582)
Supplement: Attachment — Submitted filename: Response to reviewers_2.docx [file pone.0317582.s005.docx]

**Manuscript ID:** PONE-D-23-26548

**Manuscript Title:** Prevalence, predisposing factors, and turnover intention related to low back pain among health workers in Accra, Ghana

| **Reviewer 3 comments** | **Authors’ response** | **Page**  **number** |
| --- | --- | --- |
| • Line 78: The term "neglected" is not accurate here; please consider using a different word. | The word “overlooked” has been used to replace ‘neglected’. | 3 |
| • Lines 81–83: Please revise the English for clarity. | The sentence has been revised to improve clarity. | 3 |
| • Line 92: The word “however” is not the appropriate connector here. | The word “however” has been replaced with ‘moreover’. | 3 |
| • Line 93: Consider using "the main activities" instead of "some of the…" | The phrase, “The main activities” has been used to replace the phrase, “some of these”. | 3 |
| • Lines 96–97: The sentence could be improved for clarity. Consider stating the workload intensity of these tasks first, and then, in a separate sentence, note that it is even more exhausting in Africa and other low- and middle-income countries where working aids are lacking (13, 14). | The sentence has been revised for clarity | 3 |
| • Line 125: Use "rates" instead of "rate." | The word “rates” has been used. | 4 |
| • Line 361: Replace "and" with "while" to improve flow. | The word “while” has been used to replace “and”. | 10 |
| • Lines 349 and 363: The section title and table title are somewhat vague. Consider editing them to "Occupational Factors Contributing to Lower Back Pain." | The section tile and table title have been revised. | 10 |
| • Lines 356 and 373: Clarify these titles as well; I suggest "Organizational, Behavioral, and InterventionRelated Factors Contributing to Lower Back Pain. | The section tile and table title have been revised. | 11, 12 |
| • Line 245 - Study Questionnaire and Data Collection: How were validity and reliability tested? • Language of questionnaire: In which language was the questionnaire administered? If it was originally in English, was it translated into the local language of Ghana? | As indicated in the manuscript, some portions of the questionnaire were adapted from an already validated standard questionnaire. Also, the draft questionnaire was pretested among 60 health workers to assess their validity and reliability. This is also shown in the manuscript. The manuscript has been modified to clarify how the validity and reliability were tested.  The language of questionnaire was in English since most of the health workers were literate. Moreover, already trained research assistants administered the questionnaire in the form of an interview with a few study participants who needed help in completing the questionnaire. The language of questionnaire has been specified in the manuscript. | 6  6 |
| • Table 1: Could you clarify why a majority (84%) of participants are from public hospitals? Is there a particular reason for this distribution? | The public hospitals were far more than the private ones. Also, most health workers were engaged by public hospitals. Again, hospitals were randomly (irrespective of private and public) selected in districts with more than one major facility. Respectively, please read the section on sampling procedure for more clarification. | 8-9 |
| 1. The discussion can be strengthened by explaining more and providing more supporting arguments on why ‘overtime work, on-call duties, understaffing, and working more than five days per week—are significantly associated with increased lower back pain among healthcare workers’ | The discussion section has been improved in areas related to overtime work, understaffing and working more than five days. On-call duty was not significant on the multiple logistic regression and was not discussed. | 20-24 |
| 2. Another point that should be further discussed and explained is the “Training on Proper Posture vs. Application”. In table 6, Trained on good working posture was 0.446. This may indicate a gap between training and practical application. The discussion should explore potential reasons, such as high workload, time pressure, or lack of ergonomic support, which might prevent staff from consistently applying proper posture techniques. Furthermore, the authors could consider whether reinforcement mechanisms or regular | Respectively, this is a study that employed cross-sectional study design and not cohort study design. Also, table 6 contains results of chi-square analysis and not multiple logistic regression. Moreover, even on the chi-square analysis, the variable is not significant. Therefore, it doesn’t guarantee discussion. In quantitative research, it is inappropriate to discuss a variable that is not significant (not main findings). | 15 |
| 3. Unexpected Finding: The finding that heavy lifting is not significantly associated with lower back pain is unexpected, especially given that posture training did not correlate with reduced pain. The authors should consider discussing possible explanations, such as the type or frequency of lifting tasks, use of assistive devices, … etc. 4. The authors should consider performing a statistical analysis or categorization based on the specific domain or role of healthcare staff (e.g., nurses, midwife…) to examine if lower back pain is more prevalent in certain occupational groups. This could reveal whether specific job functions contribute differently to the development of lower back pain | 3. Respectively, in research, it is inappropriate to discuss a variable that is not significant. If it was significant and unexpected, it would have been given the necessary discussion. As a rule, discussions are written for significant findings, and not any finding at all.  4. This was done in the study, but it did not show any significant difference. This is the reason why it was not shown. |  |
| Major comments for recommendations: 1. Elaborate more on the recommendations at the end of discussion section and conclusion. o Enhanced Ergonomic Training and Application: o Regular reminders, visual cues, or periodic "posture refreshers" could be used to help healthcare staff remember to implement proper techniques during work. o Ensure access to assistive lifting devices, such as mechanical lifts o Encourage healthcare institutions to foster a culture that prioritizes employee health and safety by regularly addressing occupational health risks. o Establish a reporting system where staff can communicate ergonomic concerns or report instances of musculoskeletal pain. | The discussion section has been improved with these recommendations in the last paragraph on study findings implications for policy and practice. | 24 |
